# Improvement of β‐Xylosidase and Endoxylanase Activities in *Talaromyces amestolkiae* by Genetic Manipulation of the Transcriptional Activator XlnR

**DOI:** 10.1111/1751-7915.70166

**Published:** 2025-05-26

**Authors:** Ana Pozo‐Rodríguez, Miguel Ángel Peñalva, Jorge Barriuso, Eduardo A. Espeso, María Jesús Martínez

**Affiliations:** ^1^ Microbial Systems and Protein Engineering Group, Department of Biotechnology, Center for Biological Research Margarita Salas Spanish National Research Council (CIB‐CSIC) Madrid Spain; ^2^ Aspergillus Cell Biology Group, Department of Cellular and Molecular Biosciences, Center for Biological Research Margarita Salas Spanish National Research Council (CIB‐CSIC) Madrid Spain

**Keywords:** filamentous fungi, fungal transformation, hemicellulases, xylanases

## Abstract

The ascomycete *Talaromyces amestolkiae* is a promising source of glycosyl hydrolases for hemicellulose degradation, as it contains a considerably higher number of genes encoding these enzymes than other fungi exploited for plant biomass valorisation. The development of genetic engineering tools could further improve its biotechnological potential. We report here a transformation system for *T. amestolkiae* based on pyrimidine auxotrophy complementation, which was used to successfully introduce both integrative and autonomously replicating plasmids. Then, we applied this tool to force the expression of the transcriptional activator XlnR, generating an engineered strain with enhanced β‐xylosidase (1.4‐fold) and endoxylanase (2.0‐fold) activities compared to the wild‐type cultured on xylan. Markedly larger improvements were obtained after introducing Ala788Val or Val785Phe substitutions in XlnR, achieving 3.3‐fold and 3.9‐fold increases in β‐xylosidase and endoxylanase activities, respectively, in the case of XlnR^V785F^. This recombinant strain also displays a partial deregulation of the hemicellulolytic system when cultivated on glucose and glycerol (a low‐cost and renewable substrate), yielding notably higher production of β‐xylosidases (16.9‐fold and 13.8‐fold) and endoxylanases (31.9‐fold and 22.7‐fold) than the wild‐type. Increased efficiencies of XlnR^V785F^ enzymatic crudes in xylan saccharifications showed the potential of XlnR engineering to develop robust *T. amestolkiae* strains for the valorisation of hemicellulosic residues.

## Graphical abstract

A successful transformation procedure for the genetic manipulation of the fungus *Talaromyces amestolkiae* was developed, enabling the forced expression of the transcriptional activator XlnR. A mutant version of XlnR carrying a Val785Phe substitution led to remarkably higher β‐xylosidase and endoxylanase activities compared to the wild‐type strain, which enhances the efficiency of its hemicellulolytic cocktails.

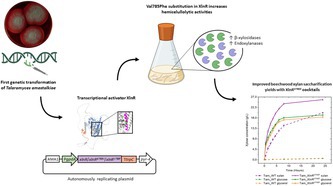

## 1 Introduction

Hemicellulose, one of the main components of plant cell walls together with cellulose and lignin, is the second most abundant renewable carbon source on Earth (Geng et al. 2022). It refers to a group of heterogeneous polysaccharides, with xylans being the predominant form, that differ in their backbone, branches, and type and distribution of glycosidic linkages (Méndez‐Líter et al. 2021a, 2021b). Several products of industrial interest can be obtained from hemicellulose such as oligosaccharides with prebiotic effect and fermentable sugars (mostly xylose) from which ethanol and xylitol, among others, can be synthesised (Méndez‐Líter et al. 2021b; Geng et al. 2022). Moreover, in plant biomass saccharification processes, the removal of hemicellulose is of great importance to increase cellulose accessibility (Meng and Ragauskas 2014).

Hemicellulose degradation can be conducted in several ways; however, the enzymatic approach is preferred over the chemical one because it is more environmentally friendly and does not generate undesirable side compounds (Ostadjoo et al. 2019). Hemicellulases are a class of glycosyl hydrolases that act on hemicellulose, and they are extensively produced by filamentous fungi. The variable composition of hemicellulose requires the concerted action of multiple hemicellulases for its degradation, being the endo‐β‐1,4‐xylanases and β‐xylosidases the most important ones (Méndez‐Líter et al. 2021b).

There is a tight regulation that controls the production of hemicellulases and typically relies on the presence of their corresponding substrate in the medium (de Eugenio et al. 2017; di Cologna et al. 2018). The most accepted model suggests that the large size of polysaccharides prevents their direct import into the cells and, in consequence, filamentous fungi need to constitutively express and/or express under carbon starvation certain hemicellulases (Benocci et al. 2017; Méndez‐Líter et al. 2021a, 2021b). These enzymes, although produced at low levels, initiate hydrolysis releasing disaccharides and monosaccharides, which enter the intracellular environment and act as inducers of the entire hemicellulolytic system via substrate‐specific transcription factors. In this sense, these factors not only control the expression of hemicellulases and other glycosyl hydrolases, but also regulate the expression levels of genes coding for specific transporters and those belonging to catabolic pathways (Benocci et al. 2017; Alazi and Ram 2018).

The ascomycete *Talaromyces amestolkiae* stands out for its remarkable production of glycosyl hydrolases (Prieto et al. 2021). Almost 200 genes encoding these enzymes have been predicted in its genome, which is a number significantly higher than those found in other fungi involved in cellulose and hemicellulose degradation, such as *Trichoderma reesei*, *Penicillium oxalicum*, or *Aspergillus niger* (de Eugenio et al. 2017). Several glycosyl hydrolases of this fungus, including 3 β‐glucosidases, 2 endo‐β‐1,4‐glucanases, 1 β‐xylosidase, 2 endo‐β‐1,4‐xylanases, and 2 α‐L‐arabinofuranosidases, have been characterised and used in saccharification processes, oligosaccharide synthesis, and glycosylation of bioactive compounds (Prieto et al. 2021). In addition, *T. amestolkiae* strains are an attractive source of natural red colourants (de Oliveira et al. 2022) and other secondary metabolites, such as meroterpenoids, isocoumarins, and benzofurans (Li et al. 2022).

Enhancing the production of hemicellulases in filamentous fungi is a very attractive approach to improve the valorisation of the hemicellulosic fraction of plant biomass. In this regard, an increase in glycosyl hydrolase production by optimising the fermentation process has recently been reported in *T. amestolkiae* (de Eugenio et al. 2024). Nonetheless, these types of fungal fermentations are often limited by the use of insoluble substrates acting as inducers and the mycelial nature of the fungus (Mattam et al. 2022). In order to further enhance hemicellulase production in this fungus, it is convenient to develop genetic manipulation tools.

Several transformation systems are available for filamentous fungi based on two types of selection markers, which are antibiotic‐resistance genes and auxotrophic complementation. Since the use of the first approach involves safety concerns about the spread of antibiotic resistant strains and there are also legal requirements from industries handling food‐related products, auxotrophic complementation is preferred (Martín 2015; Mei et al. 2019). Among this type of selection markers, pyrimidine auxotrophy is the most widely used strategy and has already been adapted successfully to some *Talaromyces* spp. (Borneman et al. 2001; Inoue et al. 2013; Delmas et al. 2014; Zhang et al. 2022). The *de novo* pathway for pyrimidine biosynthesis comprises six biochemical steps and ends up producing uridine monophosphate (UMP) (Figure S1). *pyrF* (Aleksenko et al. 1999) and *pyrG* (Oakley et al. 1987) genes, encoding orotate phosphoribosyltransferase (OPRTase) and orotidine 5′‐monophosphate decarboxylase (OMPdecase), respectively, play an essential role in this pathway. *pyrF* ^−^ and *pyrG* ^−^ mutants can be easily obtained by selecting for resistance to the antimetabolite 5‐fluoroorotic acid (5‐FOA), which is transformed by OPRTase and OMPdecase to the toxic 5‐fluoro‐UMP. These pyrimidine auxotrophs will then need the addition of uridine and/or uracil as nutritional requirements to be able to grow (Díez et al. 1987).

On the other hand, the most promising approach to improve hemicellulase production in filamentous fungi is manipulating, through genetic engineering, the transcriptional factors that regulate their expression. The main transcriptional activator of hemicellulose degradation and xylose utilisation is XlnR/XYR1/XLR1 (Van Peij et al. 1998b). This transcriptional factor contains a Zn_2_Cys_6_‐binuclear cluster domain necessary for DNA‐binding and a middle homology region with regulatory functions. The binding efficiency and the set of genes regulated by XlnR/XYR1/XLR1, as well as its role in cellulose degradation, differ between species (Benocci et al. 2017; Alazi and Ram 2018).

Several attempts have been made to enhance hemicellulase production in filamentous fungi by modulating the transcriptional regulation of XlnR/XYR1/XLR1. The first set of strategies involved the addition of extra copies of this transcriptional activator (van Peij et al. 1998a; Llanos et al. 2019) or its overexpression with constitutive strong promoters such as the ones of *gpdA* (Tamayo‐Ramos and Orejas 2014; Li et al. 2015), *tef1* (Marui et al. 2002), and *pdc1* (Wang et al. 2013) genes, which led to an improved hemicellulolytic activity. However, better results were obtained when single residue substitutions were introduced in the C‐terminal region of XlnR/XYR1/XLR1. In *P. oxalicum*, *T. reseei*, and *Neurospora crassa*, Ala871Val, Ala824Val, and Ala828Val, respectively, increased to a higher extent the production of hemicellulases (Derntl et al. 2013; Craig et al. 2015; Gao et al. 2017). In *A. niger* and *T. reseei*, Val756Phe and Val821Phe substitutions, respectively, resulted in a significant enhancement of β‐xylosidase and endoxylanase activities, even under non‐inducing conditions (Hasper et al. 2004; Ellilä et al. 2017; Kun et al. 2020; Arai et al. 2022).

This work aims to increase hemicellulase production in *T. amestolkiae* by using an approach based on the forced expression and engineering of its transcriptional factor XlnR. To achieve this goal, it was necessary to first develop a genetic modification method, which will also enable the use of this fungus in other biotechnological applications.

## 2 Experimental Procedures

### 2.1 Strains, Media and Growth Conditions

The fungus *T. amestolkiae* (hereinafter Tam_WT) was isolated from cereal wastes and deposited in the culture collection of Center for Biological Research Margarita Salas (CIB, Madrid, Spain), with reference A795. It was maintained at 28 °C in plates of potato dextrose agar (PDA, Becton Dickinson) and *Aspergillus* complete medium (MCA) (Cove 1966), which contained 1% (w/v) glucose as a carbon source and 5 mM diammonium tartrate as a nitrogen source. Cultures for *T. amestolkiae* transformation and genome sequencing were grown from conidiospores for 2–3 days in MCA liquid medium. To assess enzymatic activities and obtain crudes for saccharification studies, Tam_WT and its mutant strains (Table 1) were first cultivated on CSS medium (de Eugenio et al. 2017) from conidiospores (4.8 × 10^4^ sp/mL). After 4 days of growth, 2.5 mL of these inocula were used to start cultures on Mandels medium (de Eugenio et al. 2017) supplemented with different substrates at 1% (w/v): beechwood xylan (Megazyme), glucose (Merck) and glycerol (ITW Reagents). All liquid experiments were conducted in 250 mL flasks containing 50 mL of medium, at 28 °C and 180 rpm. 5 mM uridine and 5 mM uracil were added in plates and liquid cultures when required to complement pyrimidine auxotrophy. The conidiospores of Tam_WT and its mutant strains were preserved at −80 °C in 15% (v/v) glycerol, after determining their concentration using a Neubauer chamber.

**TABLE 1:** *Talaromyces amestolkiae* strains and plasmids used in this study.

	Description, Reference
*Fungal strain*	
Tam_WT	Wild‐type
Tam_*pyrG*14	Pyrimidine auxotroph resistant to 5‐FOA, this study
Tam_p1439^a^	Transformed with pFNO3 (p1439), this study
Tam_p1393 (colony #1)^a^	Transformed with pRG3‐AMA1‐NotI (p1393), this study
Tam_XlnR (colony #7)^a^	Transformed with p1393‐PgpdA‐XlnR‐TtrpC, this study
Tam_XlnR^A788V^ (colony #10)^a^	Transformed with p1393‐PgpdA‐XlnR^A788V^‐TtrpC, this study
Tam_XlnR^V785F^ (colony #12)^a^	Transformed with p1393‐PgpdA‐XlnR^V785F^‐TtrpC, this study
*Plasmid*	
pFNO3 (p1439)	pPV472 backbone, *amp* ^R^, *kan* ^R^, *A. fumigatus pyrG*, GlyAla5x‐GFP (Yang et al. 2004)
pRG3‐AMA1‐NotI (p1393)	pUC19 backbone, *amp* ^R^, *A. nidulans* plasmid replicator AMA1, *N. crassa pyr4* (Osherov et al. 2000)
pUC18‐PgpdA‐XlnR‐TtrpC	pUC18 backbone, *amp* ^R^, P*gpdA*::*xlnR*::T*trpC* cassette, this study, for cloning steps
p1393‐PgpdA‐XlnR‐TtrpC	P1393 derivative, P*gpdA*::*xlnR*::T*trpC* cassette, this study
pUC18‐PgpdA‐XlnR^A788V^‐TtrpC	pUC18 backbone, *amp* ^R^, P*gpdA*::*xlnR* ^A788V^::T*trpC* cassette, this study, for cloning steps
p1393‐PgpdA‐XlnR^A788V^‐TtrpC	P1393 derivative, P*gpdA*::*xlnR* ^A788V^::T*trpC* cassette, this study
pUC18‐PgpdA‐XlnR^V785F^‐TtrpC	pUC18 backbone, *amp* ^R^, P*gpdA*::*xlnR* ^V785F^::T*trpC* cassette, with this study, for cloning steps
p1393‐PgpdA‐XlnR^V785F^‐TtrpC	P1393 derivative, P*gpdA*::*xlnR* ^V785F^::T*trpC* cassette, this study

^a^ These recombinant strains were generated by transformation of Tam_*pyrG*14.

*Escherichia coli* DH5α (Invitrogen) was used for cloning and plasmid propagation and was cultivated on Lysogeny Broth (LB) medium (Bertani 1951).

### 2.2 Construction of Plasmids

All plasmids used in this work can be found in Table 1. The first attempt to transform *T. amestolkiae* was performed with the integrative plasmid pFNO3 (p1439 in our collection) (Yang et al. 2004), which contains an ATG‐less chimera composed of a repetition of 5 Gly‐Ala residues (GA5x) fused in frame to the sGFP, and the *Aspergillus fumigatus pyrG* gene as a selection marker. This plasmid is generally employed to tag with fluorescence specific genes, but in our case, its purpose was to serve as a control of integration of a non‐expressed copy of GFP in the fungus.

Next, to obtain maximum expression levels, we used the autonomously replicating plasmid pRG3‐AMA1‐NotI (p1393 in our collection) (Osherov et al. 2000) carrying the AMA1 replicator of *Aspergillus nidulans* and *N. crassa pyr4* gene as a selection marker. This vector served as a negative control of expression, and it is the backbone of the remaining p1393‐based plasmids of this work.

Another cassette, harbouring *xlnR* gene of Tam_WT (GeneBank Accesion Number BHQ10_006915, Figure S2A,B) under the control of *A. nidulans* glyceraldehyde‐3‐phosphate dehydrogenase promoter (P*gpdA*) and *A. nidulans* multifunctional tryptophan biosynthesis protein terminator (T*trpC*), was introduced in p1393 with the aim of forcing the expression of this transcriptional activator. The cassette was synthesised and cloned in the commercial pUC18 by GenScript yielding pUC18‐PgpdA‐XlnR‐TtrpC. For cloning reasons, silent mutations were added in 4 codons of *xlnR* and restriction sites were included flanking *xlnR* and the cassette (Figure S2A). p1393 and pUC18‐PgpdA‐XlnR‐TtrpC were digested with KpnI and BamHI (New England Biolabs). Then, p1393 backbone and PgpdA‐XlnR‐TtrpC cassette were purified (Qiagen) and ligated with T4 DNA ligase (Takara). The ligation mixture was transformed into chemically competent *E. coli* DH5α cells. Colonies were subjected to cPCR and the p1393‐PgpdA‐XlnR‐TtrpC plasmid of the positive clones was sequenced with the EZ‐Seq service of Macrogen.

On the other hand, pUC18‐PgpdA‐XlnR‐TtrpC was used as a template to develop two variants of the transcriptional activator by directed mutagenesis. The alanine at position 788 was substituted by a valine (Ala788Val) in one variant, and the valine at position 785 was replaced by a phenylalanine (Val785Phe) in the other variant. For the mutagenic PCR, the ExpandLong Template PCR System (Roche) was applied using the primers of Table S1. The PCR product was digested by DpnI (New England Biolabs) to hydrolyse the parental methylated DNA. After sequencing the new cassettes, the cloning procedure continued as explained before to generate p1393‐PgpdA‐XlnR^A788V^‐TtrpC and p1393‐PgpdA‐XlnR^V785F^‐TtrpC plasmids.

### 2.3 Extraction of gDNA From Fungal Strains

In order to analyse the genotype of Tam_WT and its mutant strains, gDNA was extracted from their conidiospores. Briefly, sterile wooden toothpicks were used to collect conidiospores of fresh fungal cultures grown on PDA or MCA plates. Then, conidiospores were placed on 200 μL breaking buffer containing 2% (v/v) Triton X‐100, 1% (v/v) SDS, 100 mM NaCl, 10 mM Tris–HCl pH 8 and 1 mM EDTA pH 8. Approximately 150 mg of glass beads (0.4–0.6 mm, Merck) were added to help disrupt the conidiospores. Samples were vortexed for 30 s and subsequently incubated at 70°C for 30 min. Three more vortexing steps of 30 s were performed while incubating. After that, 200 μL phenol/chloroform/isoamyl alcohol (25:24:1 v/v, Meck) were added and samples were vortexed again for 5 min. Disrupted samples were finally centrifuged at 14,000 rpm for 5 min, and 50 μL of the aqueous solution carrying the dissolved gDNA was transferred to a clean tube.

### 2.4 Isolation of 5‐FOA‐Resistant Mutants

The generation of 5‐FOA‐resistant mutants from Tam_WT was conducted in MCA plates with 2 mg/mL 5‐FOA (Apollo Scientific) and 5 mM uridine and uracil. A toothpick was heavily loaded with fresh conidiospores and used to inoculate a plate at 7 evenly distributed spots. As the toothpick was used repeatedly without reloading, the conidiospores concentration decreased in each spot, which ensures reaching the optimal conditions for the generation of 5‐FOA‐resistant sectors. 16 plates were prepared following this procedure, resulting in 112 inoculation spots, and incubated at 28 °C for up to 12 days. 5‐FOA‐resistant mutants were then purified through one selective pass on MCA plates containing the same concentrations of 5‐FOA, uracil, and uridine. Singles colonies of each mutant were subsequently tested for pyrimidine auxotrophy on MCA plates with and without uridine and uracil, since the mutants will need these two compounds to grow. To check the genotype of the mutants, gDNA was extracted as described before and used as a template for the amplification of *pyrF* and *pyrG* genes involved in pyrimidine synthesis. The PCRs were performed in 50 μL reaction mixtures that contained the PrimeSTAR GXL DNA Polymerase (Takara Bio) and specific primers for both genes (Table S1). The amplified products were purified using the QIAquick PCR purification kit (Qiagen) and finally sequenced with the EZ‐Seq service of Macrogen. Alignments of the orthologous *pyrF* and *pyrG* genes with the wild‐type ones (GeneBank accesion numbers BHQ10_006112 and BHQ10_007154, respectively) were performed to determine the position and type of acquired mutations.

### 2.5 Fungal Transformation and Selection of Transformants

The auxotroph mutant Tam_*pyrG*14 was transformed as described for *A. nidulans* (Tilburn et al. 1983), following the methodology of chemical transformation of competent protoplasts. Protoplasts were generated by resuspending 2 g of mycelium in 20 mL of solution A (1.2 M MgSO_4_·7H_2_O, 7.2 mM Na_2_HPO_4_, 2.8 mM NaH_2_PO_4_) and incubating it for 1:30 h with 600 mg of a mixture of cell wall‐degrading enzymes called Vinoflow (Novozymes). The protoplasts were then purified by equally dividing the 20 mL suspension into two 50 mL tubes. In each tube, 10 mL of solution B (0.1 M Tris–HCl pH 7.5, 0.6 M sorbitol) was gently added without mixing the two phases. The tubes were centrifuged for 10 min at 4400 × *g* and 4 °C (Eppendorf 5810R centrifuge). The protoplast band, formed at the interface, was recovered using a Pasteur pipette and transferred to a new tube, to which twice the volume of solution C (10 mM Tris–HCl pH 7.5, 1 M sorbitol) was added. After mixing by inversion, and centrifugation under the same conditions, the protoplast pellet was resuspended in 1 mL of solution C and transferred to a 1.5 mL tube. Following a 3 min centrifugation at 5000 × *g* and RT, the supernatant was discarded, and the protoplasts were resuspended again in 1 mL of solution C. This washing step was repeated two more times. In the final step, protoplasts were resuspended in 450 μL of solution D (10 mM Tris–HCl pH 7.5, 1 M sorbitol, 0.12 M CaCl_2_).

Then, protoplasts were transformed with the circular plasmids of Table 1 by applying CaCl_2_ and polyethylene glycol (PEG). In the case of plasmid p1439, 2.5 μg of DNA were used, whereas 600 ng of DNA were employed for the autonomously replicating plasmid p1393 and its derivatives. The transformation procedure was carried out in 50 mL tubes by mixing 50 μL of solution E (600 g/L PEG6000, 10 mM Tris–HCl pH 7.5, 0.12 M CaCl_2_), 40 μL of solution D, the circular plasmids dissolved in 10 μL of sterile water, and 200 μL of the protoplast suspension. A control without DNA was also prepared. The tubes were gently mixed and incubated on ice for 20 min. Subsequently, 1 mL of solution E was added to each tube, and the mixture was incubated for 5 min at RT. Finally, the volume was adjusted to 5 mL with solution D and completed to 20 mL with recovery medium (MCA with 1 M sucrose as osmotic stabiliser and without uracil and uridine) containing 0.75% (w/v) agar. The tubes were briefly mixed by inversion to embed the protoplasts, and their content distributed across 4 recovery medium plates, with1.5% (w/v) agar and without uracil and uridine, to select transformed colonies. The no‐DNA control was plated onto 2 selective recovery medium plates (negative control) and 2 plates supplemented with uridine and uracil (positive control for protoplast regeneration). Plates were incubated at 28 °C for at least 7 days.

Potential transformants were further isolated through one selective pass on MCA plates without uracil and uridine. Then, their gDNA was extracted and used to check by PCR the presence of targeted genes of the plasmids. In the case of mutants transformed with p1393 and its derivatives, an enzymatic activity screening to select the best performing mutant of each transformation was carried out.

### 2.6 Whole Genome Sequencing of Tam_XlnR^V785F^ Recombinant Strain

For sequencing the genome of Tam_XlnR^V785F^, cultures were grown on MCA liquid medium, mycelium was harvested by centrifugation, and gDNA was extracted using the NZY plant/fungi gDNA isolation kit (NZYtech). Paired‐end sequencing was then performed on an Illumina PE150 system using the services of the company Novogene, which was also in charge of removing low‐quality reads and reads that maintained adaptors using Fastp v.0.20.0 software (Chen et al. 2018). A high quality (Q30 of 92.07%) DNA library composed of 17,434,018 clean reads of 150 bp in length was received. Finally, the Bioinformatics and Biostatistics facility of the CIB analysed the sequencing data to determine the behaviour of p1393‐PgpdA‐XlnR^V785F^‐TtrpC plasmid in Tam_XlnR^V785F^. With this purpose, the clean reads were mapped using the BWA/0.7.17 aligner (Li and Durbin 2009) against the scaffolds of *T. amestolkiae* genome (GenBank Accession Number ASM189636v1) and the sequence of the plasmid. Further studies were conducted with SAMtools/1.14 (Danecek et al. 2021) and R/4.3.2 (https://www.R‐project.org) softwares.

### 2.7 Enzyme Activity and Protein Assays

To evaluate the enzymatic activities and extracellular protein concentration of Tam_WT, Tam_*pyrG*14, Tam_p1393, Tam_XlnR, Tam_XlnR,^A788V^ and Tam_XlnR^V785F^, they were grown on Mandels medium supplemented with 1% (w/v) xylan, glucose, or glycerol. Samples were taken daily and centrifuged for 10 min at max. speed and 4 °C to retrieve the supernatants.

β‐xylosidase activity was determined spectrophotometrically at 410 nm by the release of 4‐nitrophenol (*p*NP) (*ε*_410_ = 15,200 M^−1^ cm^−1^) using 0.1% (w/v) *p*‐nitrophenyl‐β‐d‐xylopyranoside (*p*NPX, Sigma‐Aldrich) in 50 mM sodium acetate buffer pH 5. Reactions (200 μL) were incubated for 10 min at 50 °C and 1200 rpm and stopped by changing the pH through the addition of 500 μL of 2% (w/v) Na_2_CO_3_. Endoxylanase activity was calculated from the hydrolysis of 3% (w/v) beechwood xylan (Megazyme) dissolved in 50 mM sodium acetate buffer pH 5. Reactions (500 μL) were incubated for 10 min at 50 °C and 1200 rpm and stopped by heating at 100 °C for 5 min. After centrifuging for 5 min at 20,000 × *g* and RT, the release of reducing sugars was measured spectrophotometrically at 540 nm using the Somogyi‐Nelson method (Somogyi 1945). One unit of activity was defined as the amount of enzyme capable of releasing 1 μmol of *p*NP or reducing sugars per minute. In the initial screenings conducted to select the best performing transformants, endoxylanase activity was determined using Azo‐Xylan Birchwood Powder (Megazyme) following the manufacturer's protocol.

Extracellular protein concentration was quantified utilising the Bradford dye reagent (Bio‐Rad), based on the original Bradford method (Bradford 1976), and Bovine Serum Albumin (BSA, Thermo Fisher Scientific) as the standard. Measurements were performed with a spectrophotometer at 595 nm.

For SDS‐PAGE and zymograms, enzymatic crudes were concentrated 10× and dialysed against 100 mM sodium acetate buffer pH 5 in Amicon Ultra—0.5 mL 3 kDa devices (Merck). Crudes (14 μg of extracellular proteins) were heated up at 98 °C for 10 min in a buffer containing SDS and β‐mercaptoethanol before being loaded into 10% polyacrylamide gels. Precision Plus Protein Dual Colour Standard (Bio‐Rad) was the molecular weight marker employed, and proteins were stained with Coomassie Brilliant Blue R‐250 (Bio‐Rad). For endoxylanase zymograms, 0.2% (w/v) beechwood xylan was incorporated into SDS‐PAGE 10% polyacrylamide gels, and 1.5 μg of extracellular proteins from the crudes were loaded. After electrophoresis, gels were washed in 5% (v/v) Triton X‐100 solution for 1 h to remove SDS and incubated in distilled water for 30 min at 30 °C to set the conditions for endoxylanase activity. Then, gels were stained with 0.1% (w/v) Congo Red for 30 min and washed with 1 M NaCl until activity bands became visible. Gels were finally immersed in 5% (v/v) acetic acid to turn the background black. For β‐xylosidase zymograms, isoelectric focusing was carried out on 5% polyacrylamide gels using pH 3–10 ampholytes (GE Healthcare), with 1 M H_3_PO_4_ and 1 M NaOH as anode and cathode buffers, respectively. 1 μg of extracellular proteins were loaded for each crude. The pH gradient was directly measured on the gels with a contact electrode (Crison). β‐Xylosidase activity was detected by incubating the gels with 2 mM *p*‐methylumbelliferyl‐β‐d‐xylopyranoside (Sigma‐Aldrich) for 10 min at 30°C, and fluorescence was visualised under UV light using a Gel Doc XR+ system (Bio‐Rad).

### 2.8 Biomass Determination

Tam_WT, Tam_p1393, Tam_XlnR, Tam_XlnR^A788V^ and Tam_XlnR^V785F^ growth in liquid culture was compared by estimating ergosterol content, which is a specific sterol of fungal cell walls (Ng et al. 2008). 2 mL samples were centrifuged (10 min at max. speed and 4 °C) and lyophilized. The pelleted biomass was transferred to 8 mL KIMAX tubes (DWK Life Sciences) and dissolved in 200 μL petroleum ether (Merck) and 800 μL 10% (w/v) KOH (Merck) solubilised in methanol (Merck). Then, samples were sonicated for 15 min in an ultrasound bath and, after 45 min at RT, incubated at 70 °C for 90 min. Once cooled down, 0.4 g/L cholesterol (Merck) was added as an internal standard, together with 200 μL water and 400 μL petroleum ether for ergosterol and cholesterol extraction. Samples were mixed with a 30 s vortex step and let them stand until the phases were separated. The organic phase, containing petroleum ether together with ergosterol and cholesterol, was placed in a 2 mL tube and dried by evaporation at 25 °C to repeat one more time the extraction. Finally, the extracted samples were resuspended in 400 μL methanol, mixed and filtered through 0.22 μm PTFE syringe filters (Agilent).

The detection and quantification of ergosterol and cholesterol was carried out by HPLC in an Agilent 1200 series LC instrument equipped with a reverse phase ZORBAX Eclipse plus C18 column (Agilent). The mobile phase was a mixture of methanol and acetonitrile (80:20, v/v), at a flow rate of 1 mL/min, and the volume of sample injected was 10 μL. Ergosterol and cholesterol peaks were detected at 254 and 204 nm, respectively, and quantified by comparing their areas with the calibration curves of each compound prepared in methanol. Ultimately, fungal biomass was estimated from the ergosterol content using a calibration curve plotting ergosterol content per gram of lyophilized Tam_WT biomass.

### 2.9 Saccharification Assays

Tam_WT and Tam_XlnR^V785F^ cultures, grown on Mandels medium supplemented with xylan, glucose and glycerol, were centrifuged for 10 min at maximum speed and 4 °C when highest levels of enzymatic activities were reached. Then, the supernatants were sequentially vacuum filtered through 0.8, 0.45 and 0.22 μm nitrocellulose membrane discs (Millipore) to obtain the enzymatic crudes.

Saccharification reactions were performed in 2 mL tubes. Each reaction was composed of 450 uL of filtered enzymatic crudes mixed with 50 mM sodium acetate buffer pH 5 containing 5% (w/v) beechwood xylan (Megazyme) in a final volume of 1 mL. The reactions were incubated at 50 °C and 1200 rpm for 24 h. Samples were taken periodically, and the xylose released was immediately quantified using the D‐Xylose Assay Kit (Megazyme).

### 2.10 Bioinformatic Analyses of *pyrG* , *pyrF* and XlnR

The nucleotide sequences of *T. amestolkiae pyrG*, *pyrF*, and *xlnR* were retrieved from the genomic information available under the GenBank Accession Number ASM189636v1. The alignment of wild‐type *pyrF* and *pyrG* with their corresponding mutated homologues was performed with Snapgene software (www.snapgene.com). The identification of A788 and V785 residues in XlnR was conducted by alignment using Clustal Omega (Madeira et al. 2024) with the orthologous genes of *P. oxalicum* (Gao et al. 2017), *N. crassa* (Craig et al. 2015) and *T. reseei* (Derntl et al. 2013), in which the effect of these two substitutions had been described. The structure of *T. amestolkiae*'s OMPdecase and XlnR was predicted with AlphaFold (Jumper et al. 2021) and analysed utilising Chimera (Pettersen et al. 2004). The different domains of XlnR were identified with InterProScan (Jones et al. 2014).

### 2.11 Statistical Tests

Activity measurements to compare Tam_WT and its mutant strains were carried out employing biological triplicates and technical duplicates. Statistical differences were determined with the Student's *t*‐test (*n* = 3, *p < 0.05 and **p < 0.01).

## 3 Results and Discussion

### 3.1 Isolation of *T. amestolkiae pyr* ^−^ Mutants Resistant to 5‐FOA

Genetic transformation of *T. amestolkiae* strains had not been reported yet. A widely used method in filamentous fungi, based on the complementation of pyrimidine auxotrophs (Martín 2015; Mei et al. 2019), was chosen for this work. For that, auxotrophic mutations in the *de novo* pyrimidine biosynthetic pathway were selected using resistance to 5‐FOA (Díez et al. 1987). The optimal 5‐FOA concentration preventing growth of the fungus was determined to be 2 mg/mL on MCA. Sectors resistant to 5‐FOA were obtained by incubating wild‐type colonies for 5 to 12 days at 28 °C. 14 putative *pyr* ^−^ sectors were selected and the corresponding strains purified by plating isolated colonies derived from conidiospores. These strains were further confirmed to be uridine/uracil auxotrophs (Figure S3A). Their genotypes were determined after PCR amplification and Sanger sequencing of the coding regions of *pyrF* (785 bp long) and *pyrG* (887 bp long), which encode the enzymes catalysing the two final steps of the *de novo* pyrimidine biosynthetic pathway (Figure S1). In all cases, convincing causative mutations in either *pyrG* or *pyrF* were detected. These mutations are listed in Table 2.

**TABLE 2:** List of *pyr* ^−^ mutants of *Talaromyces amestolkiae* isolated in this work through the acquisition of 5′‐fluoroorotic acid (5‐FOA) resistance.

Mutant	Mutation type	Nucleotide change	Protein change
*pyrF*1	Nonsense	c.C606T	Early stop codon after E183
*pyrF*2	Frameshift	c.Δ(A421 → C458)	Truncation after N122
*pyrG*3	Frameshift	c.Δ(G720 → A741)	Truncation after D223
*pyrF*4	Deletion of 3 bp	c.Δ(C506 → T508)	Deletion of V151
*pyrG5*	Deletion of 18 bp	c.Δ(A558 → T575)	Deletion of T170‐D175
*pyrF*6	Frameshift	c.ΔA549	Truncation after I164
*pyrF*7	Missense	c.G3A	Met1Ile, loss start codon^a^
*pyrF*8	Missense	c.T2C	Met1Thr, loss start codon^a^
*pyrF10*	Frameshift	c.117insT	Truncation after F40
*pyrG11*	Deletion of 12 bp	c.Δ(G822 → G833)	Deletion of A258‐Q261
*pyrF13*	Missense	c.T2C	Met1Thr, loss start codon^a^
*pyrG14*	Frameshift	c.Δ(C144 → G154)	Truncation after L48
*pyrF15*	Frameshift	c.136insT	Truncation after L46
*pyrG16*	Frameshift	c.279insA	Truncation after T76

^a^ In *Talaromyces amestokiae pyrF*, the next start codon is out of frame.

The most frequent mutations are deletions, while substitutions and insertions are less represented. Except in three strains, these mutations caused premature truncations of the protein or resulted in early stop codons or loss of the start Met codon. Therefore, these data strongly indicate that these mutations lead to a complete loss‐of‐function. Indeed, all the mutants were strictly dependent on supplementation with uridine and uracil for their growth. *T. amestolkiae pyrG*14 (Figure S3B, hereinafter Tam_*pyrG*14), resulting in the earliest frameshift in *pyrG* (removing the 231 C‐terminal residues of the 278 full length OMPdecase), was chosen for subsequent work. The deleted residues form part of an α‐helix located upstream of the main catalytic residues of the enzyme (Traut and Temple 2000) (Figure S4), strongly suggesting that *pyrG*14 is a null allele. Deletion of 11 pb of the coding region near the codons of catalytic site residues would help prevent the isolation of spontaneous *pyrG* ^+^ revertants during selection of transformants. These results, in combination with previous reports on *T. marneffei* (Borneman et al. 2001), highlight the utility of 5‐FOA to select pyrimidine auxotrophs in *Talaromyces* species.

### 3.2 Transformation of *T. amestolkiae* With an integrative plasmid by pyrimidine auxotrophy complementation

**FIGURE 1:**
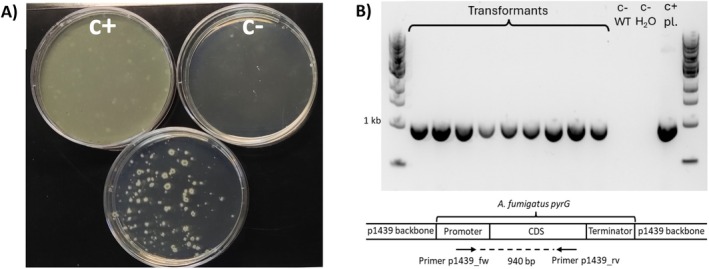
*Talaromyces amestolkiae* mutants transformed with the integrative plasmid p1439 (pyrimidine auxotrophy complementation), yielding Tam_p1439. (A) Transformation plate together with a negative control (C−, no DNA) and positive control for protoplast regeneration (C+, no selection). (B) Genotyping PCR to check the presence of *Aspergillus fumigatus pyrG* gene in nine Tam_p1439 transformants. A positive control with p1439 plasmid and negative controls with Tam_WT and water were included. The expected amplification size was 940 bp. 1 kb DNA Ladder NEB was employed.

Once a pyrimidine auxotroph of *T. amestolkiae* was obtained, we used PEG/calcium‐mediated transformation of protoplasts following the protocol described for *A. nidulans* (Tilburn et al. 1983). *Talaromyces amestolkiae* was incubated with different concentrations of Vinoflow enzymes and for different times. A mycelial mass of 2 g and an incubation time of 1 h and 30 min with 600 mg of Vinoflow were optimal to generate viable protoplasts able to grow on regeneration medium containing 1 M sucrose as osmotic stabiliser. These protoplasts were transformed with the integrative plasmid pFNO3 (p1439 in our collection), which carries the *A. fumigatus pyrG* gene that complements Tam_*pyrG*14 pyrimidine auxotrophy, and will enable random integration of foreign DNA into *T. amestolkiae*'s genome. Numerous transformants showing vigorous growth on regeneration medium lacking uridine and uracil were obtained (Figure 1A). Of these, nine (Tam_p1439 #1‐9) were isolated, purified to homokaryosis and PCR‐genotyped. The results showed that all the isolates had incorporated the transforming DNA into their genomes (Figure 1B), confirming that *T. amestokiae* can be successfully transformed using this methodology. In addition to avoiding the use of antibiotic resistance genes, complementation of pyrimidine auxotrophs with a homologous *pyrG* gene offers other advantages. First, the marker can be recycled using counterselection with 5‐FOA, enabling the use of the same strain for successive rounds of transformation (d'Enfert 1996), and secondly, it allows the targeting of transgenes to the *pyrG* locus (Gutiérrez et al. 1999) by homologous recombination.

### 3.3 Forced Expression of the Transcriptional Activator XlnR and Its Mutant Variants Resulted in Increased Hemicellulase Activities

The establishment of a transformation protocol for *T. amestolkiae* enabled the engineering of hemicellulase production through genetic manipulation. We introduced the autonomously replicating plasmid pRG3‐AMA1‐NotI (p1393 in our collection) (Osherov et al. 2000) carrying a transgene in which *T. amestolkiae*'s transcriptional activator XlnR was expressed under the control of the constitutive *A. nidulans* P*gpdA* promoter, resulting in plasmid p1393‐PgpdA‐XlnR‐TtrpC. This increases the copy number of the transgene (the average copy number of the extrachromosomal AMA1 element is 10–30; Aleksenko and Clutterbuck 1997) and allows its expression independently of the presence of inducing carbon sources. Other systems to introduce expression cassettes, aside from autonomously replicating plasmids, include random integration into the genome, targeted insertion at a specific locus via homologous recombination, or CRISPR‐Cas, which is very useful for fungi whose genomes are difficult to edit (S. Wang et al. 2017).

In addition to wild‐type XlnR, two mutant versions of the regulator were expressed, based on reports that these mutants increased expression of hemicellulolytic genes in *P. oxalicum*, *N. crassa* and *T. reesei* (Derntl et al. 2013; Craig et al. 2015; Ellilä et al. 2017; Gao et al. 2017; Arai et al. 2022). These XlnR versions harbour either Ala788Val or Val785Phe substitutions (amino acid numbering is for *T. amestolkiae*; these residues are conserved among XlnR homologues), yielding plasmids p1393‐PgpdA‐XlnR^A788V^‐TtrpC and p1393‐PgpdA‐XlnR^V785F^‐TtrpC, respectively.

Tam_*pyrG14* was transformed with these three plasmids and 6–8 colonies that complemented the pyrimidine auxotrophy were isolated from each transformation. In addition, the empty p1393 vector was also transformed to study the effect that this autonomously replicating plasmid has on fungal growth and to discard the unlikely possibility that the vector on its own was responsible for increasing hemicellulase production. Tam_XlnR, Tam_XlnR^A788V^, Tam_XlnR,^V785F^ and Tam_p1393 transformants were analysed on 50 mL Mandels medium supplemented with 1% (w/v) beechwood xylan as inducer, to select the best β‐xylosidase and endoxylanase producer (Figures S5–S8) using Tam_WT as control, and Tam_*pyrG*14 to confirm the lack of effect of the pyrimidine auxotrophy. The transformants displaying the highest activity values, which were Tam_XlnR #7, Tam_XlnR^A788V^ #12, Tam_XlnR^V785F^ #10, and Tam_p1393 #1, were chosen for further studies.

**FIGURE 2:**
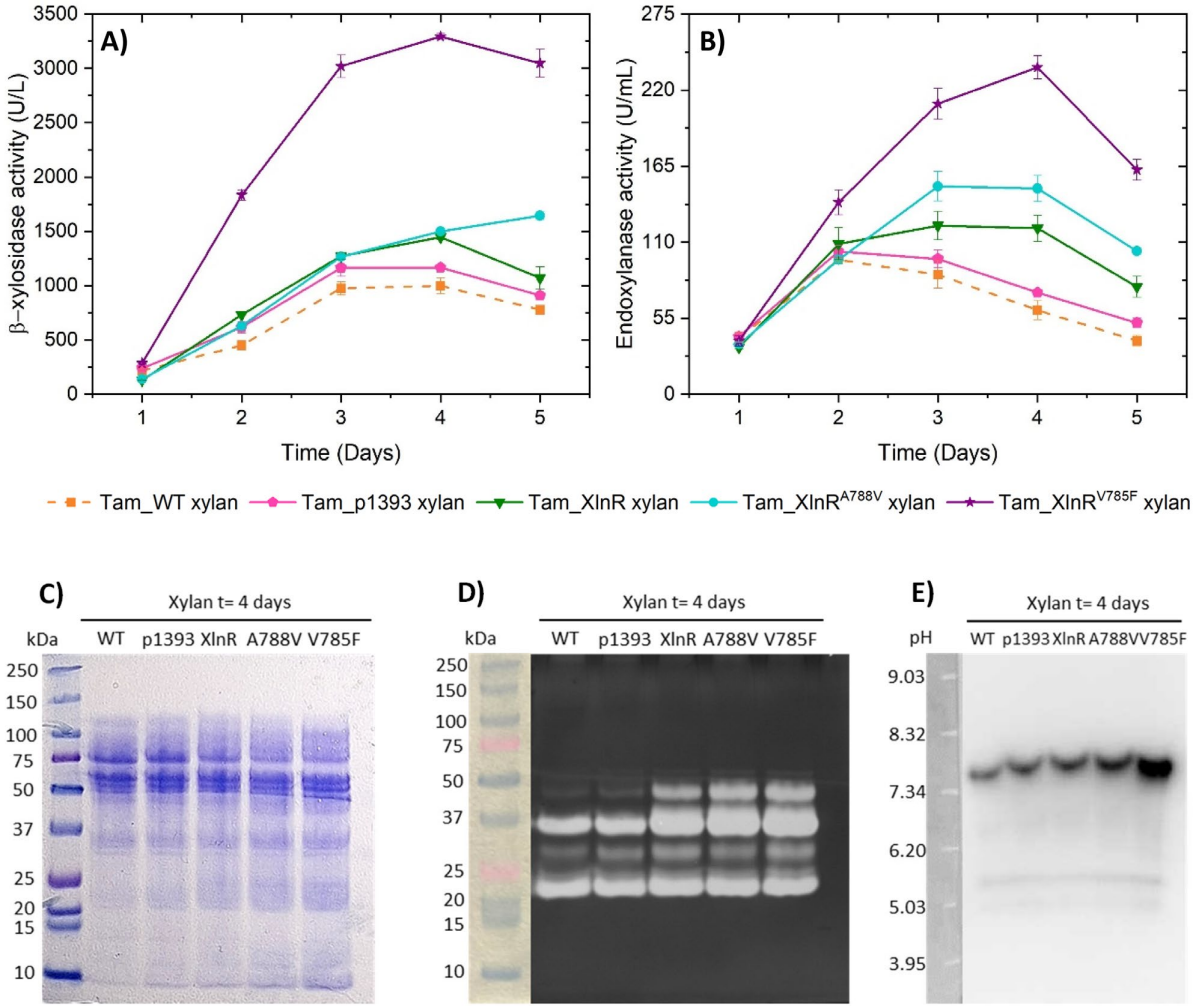
Enzymatic activities and proteins of the supernatants from Tam_WT, Tam_p1393, Tam_XlnR, Tam_XlnR^A788V^ and Tam_XlnR^V785F^ when grown on Mandels medium supplemented with 1% (w/v) beechwood xylan. Time course of (A) β‐xylosidase and (B) endoxylanase activities in fungal cultures. Error bars represent standard deviations of three biological replicates. (C) SDS‐PAGE analysis of secreted proteins. (D) Endoxylanase zymogram after SDS‐PAGE and (E) β‐xylosidase zymogram after isoelectric focusing. Concentrated crudes from day 4 of cultivation were assayed using equal extracellular protein concentrations.

These transformants and Tam_WT were cultured on 50 mL Mandels medium with 1% (w/v) beechwood xylan. β‐xylosidase and endoxylanase activities, extracellular protein concentration, and fungal growth were measured daily. Figure 2A,B, reveals that Tam_XlnR, Tam_XlnR,^A788V^ and Tam_XlnR^V785F^ mutants increased both β‐xylosidase and endoxylanase activities compared to Tam_WT and Tam_p1393, but to a different extent. When calculating the fold change in enzymatic activity of the mutants relative to Tam_WT on day 4 of cultivation, it became evident that the improvement in Tam_XlnR is not very pronounced, showing 1.4‐fold and 2.0‐fold increments in β‐xylosidase and endoxylanase activities, respectively. This suggests that forced expression of *xlnR* does not lead to a major increase in β‐xylosidase and endoxylanase activities.

While similar results have been reported with *T. reesei* (Mach‐Aigner et al. 2008; Wang et al. 2013), *P. oxalicum* (Li et al. 2015), *Penicillium canescens* (Serebryanyi et al. 2006) and *Fusarium oxysporum* (Calero‐Nieto et al. 2007), other reports concluded that *xlnR* overexpression in *A. nidulans* (Tamayo‐Ramos and Orejas 2014), *Aspergillus oryzae* (Noguchi et al. 2009), *A. niger* (Ballmann et al. 2019) and *Myceliophthora thermophila* (Wang et al. 2015) significantly increased the production of these enzymes. However, the different substrates, culture conditions, genetic engineering tools and measuring techniques preclude meaningful comparison (Llanos et al. 2019).

Tam_XlnR^A788V^ transformant showed an increase of 1.5‐fold for β‐xylosidase activity and of 2.4‐fold for endoxylanase activity on day 4 of cultivation, while in Tam_XlnR^V785F^ β‐xylosidase activity increased 3.3‐fold and endoxylanase activity 3.9‐fold. In addition, measurements of extracellular protein concentration revealed that Tam_XlnR^A788V^ and especially Tam_XlnR^V785F^ secreted more protein than the rest of the strains (Figure S9A). Therefore, introducing single residue substitutions in specific regions of XlnR is a more effective approach to improve hemicellulase production than the sole forced expression of this transcriptional activator. In this regard, in *T. amestolkiae*, Val785Phe has a larger effect than Ala788Val.

Comparison between a wild‐type strain and these three *XlnR* transformants in a single experiment has not been reported yet. In *P. oxalicum*, *xlnR* overexpression during growth on wheat bran showed minimal increases in β‐xylosidase and endoxylanase activities and in extracellular protein concentration compared to the significant improvement observed with the XlnR^A871V^ mutant. In line with the data from Tam_XlnR^V785F^, this enhancement was particularly high in the late phases of cultivation (Gao et al. 2017). Nevertheless, the alternative mutation XlnR^V868F^ has not been studied in *P. oxalicum*. In the case of *T. reesei*, equivalents of these three strains have been assayed under varying conditions, which makes direct comparison between them unreliable (Derntl et al. 2013; Ellilä et al. 2017; Lv et al. 2023; Zhao et al. 2023).

In order to determine if this improvement of β‐xylosidase and endoxylanase activities alters the pattern of proteins secreted by *T. amestolkiae*, a SDS‐PAGE analysis was carried out using equal extracellular protein concentrations of crudes at day 4 of cultivation (Figure 2C). Results revealed a very similar protein pattern across all strains. Given the complexity of *T. amestolkiae* crudes, which contain a diverse mixture of proteins, directly identifying β‐xylosidases and endoxylanases from the gel is challenging. To overcome this limitation, zymograms were conducted for these enzymatic activities. The endoxylanase zymogram was performed employing a SDS‐PAGE gel and displayed four bands, with the two larger ones being more intense in Tam_XlnR, Tam_XlnR,^A788V^ and Tam_XlnR^V785F^ mutants than in the wild‐type and p1393 control (Figure 2D). The β‐xylosidase zymogram was carried out using an isoelectric focusing gel, since BxTW1, a characterised enzyme from *T. amestolkiae* with this activity (Nieto‐Domínguez et al. 2015), is a dimeric protein that would be difficult to renature after SDS‐PAGE. This zymogram showed a single band of higher intensity in Tam_XlnR^V785F^ compared to those in the other strains (Figure 2E). These findings suggest that the enhancement of hemicellulase activities results from the differential abundance of specific β‐xylosidases and endoxylanases, rather than an overall distinct protein secretion pattern.

**FIGURE 3:**
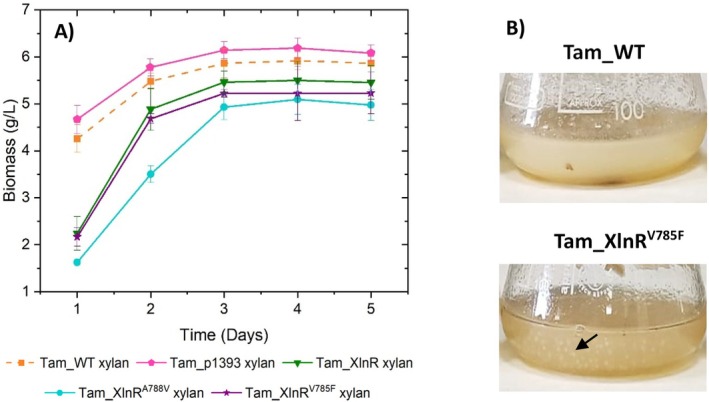
(A) Biomass concentration derived from ergosterol content of Tam_XlnR, Tam_XlnR^A788V^, Tam_XlnR^V785F^ and Tam_p1393 compared to Tam_WT when grown on Mandels medium supplemented with 1% (w/v) beechwood xylan. Error bars represent standard deviations of three biological replicates. (B) Phenotype observations of Tam_WT (Tam_p1393 showed the same aspect) and Tam_XlnR^V785F^ (Tam_XlnR and Tam_XlnR^V785F^ exhibited the same appearance) after 1 day of growth on Mandels medium supplemented with 1% (w/v) beechwood xylan.

Fungal growth was monitored by measuring ergosterol content and correlating it with biomass concentration. Figure 3A shows that Tam_XlnR, Tam_XlnR,^A788V^ and Tam_XlnR^V785F^ grow slower than Tam_WT and Tam_p1393, with these differences being more pronounced on day 1 of cultivation and becoming less noticeable as time progresses. This observation is important because it demonstrates that more hemicellulases are being produced despite diminished biomass. The negative effect on growth caused by forced expression of *xlnR* and its mutant variants is likely due to a metabolic burden associated with increased synthesis of this transcriptional activator. The higher hemicellulase production observed in the Tam_XlnR^V785F^ recombinant strain does not seem to worsen this metabolic burden since no substantial differences in growth behaviour can be seen when compared with Tam_XlnR. Forced expression of *xlnR* and its derivatives also changes mycelial morphology, as depicted in Figure 3B: Tam_WT and Tam_p1393 grow in a dense filamentous form with homogeneously dispersed hyphae, while with Tam_XlnR, Tam_XlnR,^A788V^ and Tam_XlnR^V785F^ spherical pellets resulting from hyphal aggregation are observed. These differences in fungal morphology would need to be taken into account when scaling up hemicellulase production because they can greatly affect the performance of the process (Veiter et al. 2018).

Lastly, there were no major disparities between Tam_p1393 and Tam_WT in any of the parameters measured. Thus, the autonomously replicating plasmid itself is not responsible for any enhancement in hemicellulase activity or for differences in growth among the XlnR transformants. This is in agreement with findings in *Penicillium rubens* , where a derivative of this autonomously replicating plasmid had minimal impact on growth rate, colony morphology, and biocontrol efficacy (Villarino et al. 2018).

In light of all these results, it was decided to further characterise the Tam_XlnR^V785F^ recombinant strain. By whole genome sequencing, we confirmed that the plasmid was autonomously replicating since no integration events were detected. Thus, all the phenotypic effects previously described should be attributed to the transgenes amplified with the vector, and not to any disturbance in *T. amestolkiae* genome.

### 3.4 Partial Deregulation of Hemicellulase Production in Tam_XlnR^V785F^ Transformant When Using Glucose and Glycerol as Carbon Sources

**FIGURE 4:**
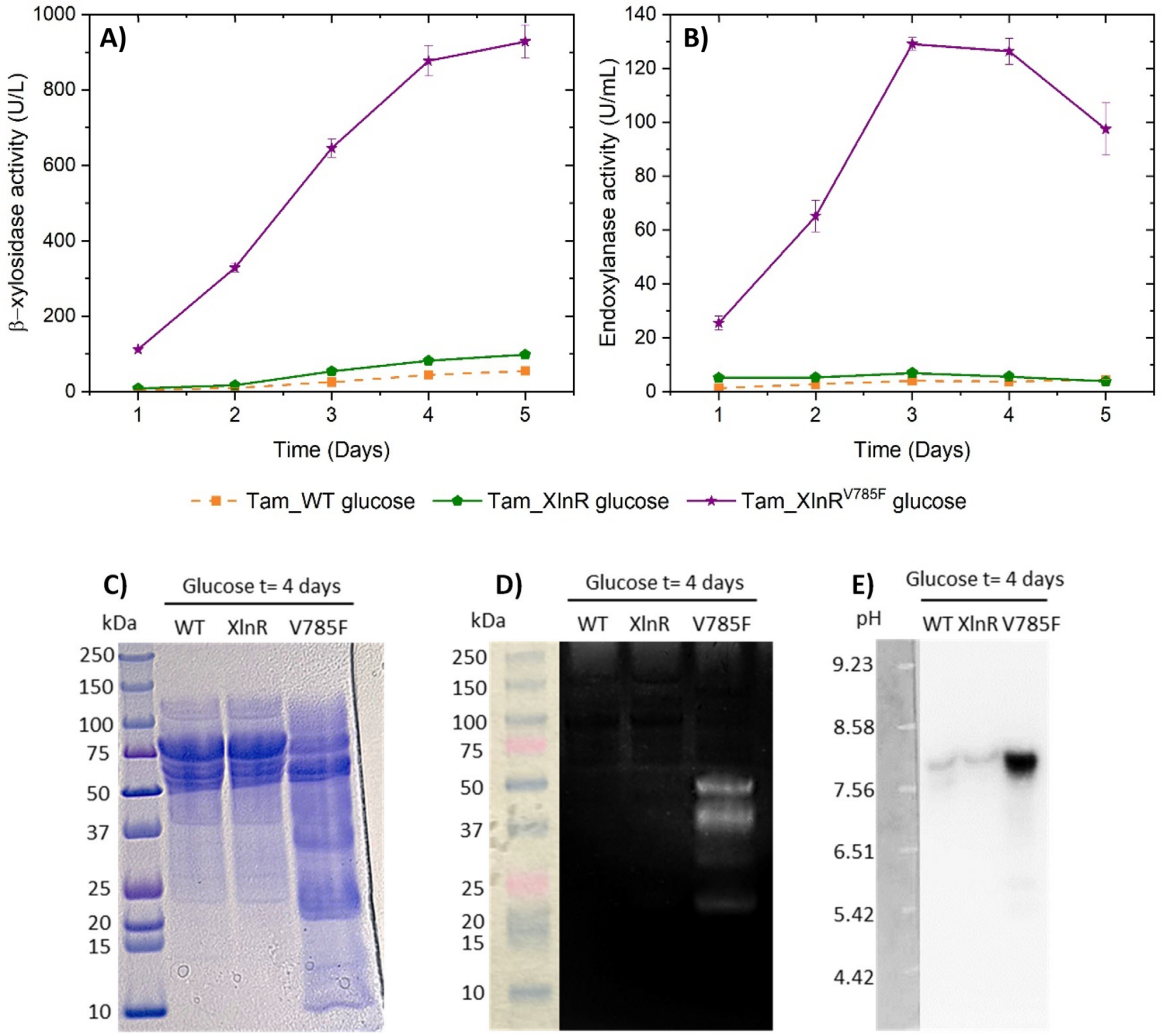
Enzymatic activities and proteins of the supernatants from Tam_WT, Tam_XlnR and Tam_XlnR^V785F^ when grown on Mandels medium supplemented with 1% (w/v) glucose. Time course of (A) β‐xylosidase and (B) endoxylanase activities. Error bars represent standard deviations of three biological replicates. (C) SDS‐PAGE analysis of secreted proteins. (D) Endoxylanase zymogram after SDS‐PAGE and (E) β‐xylosidase zymogram after isoelectric focusing. Concentrated crudes from day 4 of cultivation were assayed using equal extracellular protein concentrations.

To evaluate the production of hemicellulases by Tam_XlnR and Tam_XlnR^V785F^ under non‐inducing and carbon repressing conditions, they were cultured together with Tam_WT on 50 mL Mandels medium supplemented with 1% (w/v) glucose. Figure 4A,B shows that Tam_XlnR^V785F^ is able to secrete high amounts of β‐xylosidases and endoxylanases while barely any activity is detected in Tam_WT. Specifically, a 16.9‐fold improvement in β‐xylosidase activity on day 5 of cultivation and a 31.9‐fold increase in endoxylanase activity on day 3 of growth were observed. This reveals an important partial or complete deregulation of the hemicellulolytic system in this recombinant strain, which is capable of circumventing the strong inhibition that glucose exerts in the wild‐type by carbon catabolite repression. Regarding extracellular protein concentration, Tam_XlnR^V785F^ maintained it below that of Tam_WT at the beginning of cultivation, until it surpassed it on day 4 (Figure S9B). However, this final increase in extracellular protein concentration was markedly reduced in Tam_XlnR, as well as the enhancement of β‐xylosidase and endoxylanase activities, reaching fold increases of only 1.8 and 1.7 at days 5 and 3 of cultivation, respectively. The modest improvement in hemicellulase production by Tam_XlnR, compared to Tam_XlnR^V785F^, strongly suggests that the deregulation is not attributable to forced expression of the gene encoding the transcriptional activator XlnR, but rather to the Val785Phe substitution.

Comparable outcomes were obtained with the sole *xyr1* overexpression in *T. reesei* when grown on glucose and glycerol (Derntl et al. 2019). In agreement with our results, Ala824Val and Val821Phe substitutions in *T. reesei* XYR1 and Val756Phe in *A. niger* XlnR also enable high hemicellulase production on media supplemented with glucose or sugar beet pulp (Derntl et al. 2013; Kun et al. 2020; Zhao et al. 2023). Structural prediction of *T. amestolkiae* XlnR indicates that Val785 is part of a α‐helix located in the C‐terminal part of the so‐called middle homology region (Figure S2C). Although the exact mechanism needs to be elucidated, this region seems to be involved in the regulation of XlnR activity. Some authors have postulated that Val785Phe mutation and its alanine‐to‐valine counterpart significantly change the regulatory activity of the middle homology region by disturbing a putative inhibitory domain that typically renders this transcription factor inactive under repressing conditions, or by increasing the ability of XlnR to recruit the transcription initiation complex (Hasper et al. 2004; Xia et al. 2022).

On the other hand, the SDS‐PAGE analysis shows larger differences in the intensities of low molecular weight bands between Tam_XlnR^V785F^ and both Tam_XlnR and Tam_WT, compared to the xylan crudes (Figure 4C). This is in line with the observed results in the zymograms (Figure 4D,E), where the β‐xylosidase band is barely detectable in Tam_XlnR and Tam_WT, and the four endoxylanase bands are not visible at all. Consistent with the enzymatic activity data, these observations suggest that the Val785Phe substitution in XlnR enables high β‐xylosidase and endoxylanase production on glucose, since the corresponding bands are present in the mutant. However, there are also variations in the intensities of Tam_XlnR^V785F^ bands in the endoxylanase zymograms between the xylan (Figure 2D) and glucose (Figure 4D) crudes. Specifically, the enzymes corresponding to the two lower molecular weight bands are more difficult to detect in the glucose crudes, with this disparity being more evident for the 20 kDa band. This finding indicates that the regulation of hemicellulase production is not completely overridden with the Val785Phe mutation. The most likely explanation for this phenomenon is that, besides XlnR, several other transcription factors are involved in the regulation of the hemicellulolytic genes, and they may act differently depending on the substrate used (Tani et al. 2014; Llanos et al. 2019). For example, the transcriptional activators TctA in *Talaromyces cellulolyticus*, and ACE2 and ACE3 in *T. reesei*, have been shown to control hemicellulase production (Benocci et al. 2017). There are other transcription factors, such as CreA/Cre1/Cre‐1 in most fungi, ACE1 and XPP1 in *T. reesei* and HCR‐1 in *N. crassa* , that function as repressors of the hemicellulolytic system (Benocci et al. 2017). Furthermore, another layer of regulation related to changes in chromatin structure and promoter accessibility has been described in response to induction signals, like the presence of certain substrates (Benocci et al. 2017; Alazi and Ram 2018).

**FIGURE 5:**
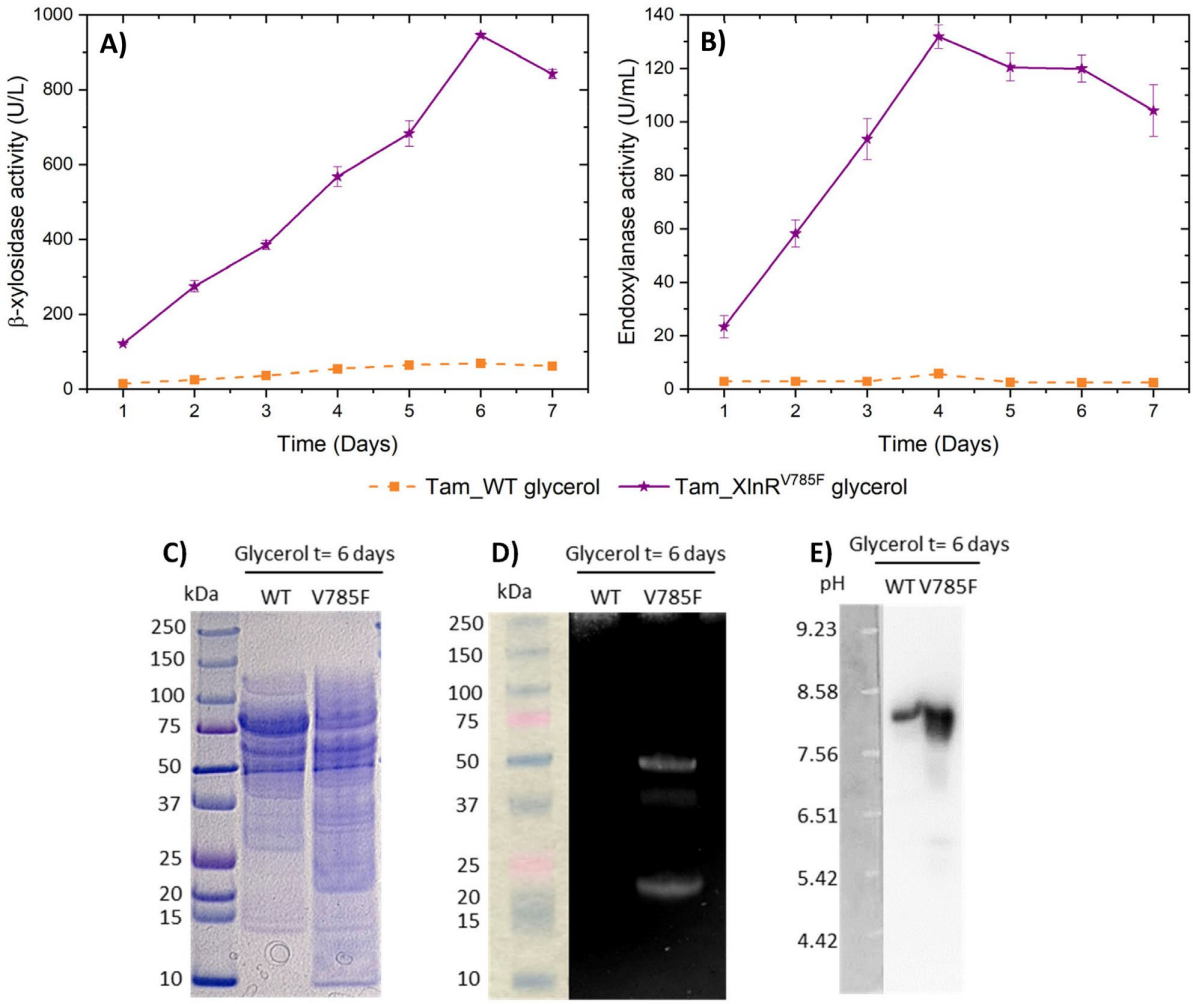
Enzymatic activities and proteins of the supernatants from Tam_WT and Tam_XlnR^V785F^ when grown on Mandels medium supplemented with 1% (w/v) glycerol. Time course of (A) β‐xylosidase and (B) endoxylanase activities. Error bars represent standard deviations of three biological replicates. (C) SDS‐PAGE analysis of secreted proteins. (D) Endoxylanase zymogram after SDS‐PAGE and (E) β‐xylosidase zymogram after isoelectric focusing. Concentrated crudes from day 6 of cultivation were assayed using equal extracellular protein concentrations.

*T. amestolkiae* has been demonstrated to be an excellent producer of robust and efficient glycosyl hydrolase cocktails on media with different carbon sources, including low‐cost and renewable substrates (Méndez‐Líter et al. 2021a; Prieto et al. 2021). In this sense, glycerol is an abundant side‐product generated from biodiesel production that could be used to obtain hemicellulases (Monteiro et al. 2018). To test this, Tam_XlnR^V785F^ and Tam_WT were grown on 50 mL Mandels medium supplemented with 1% (w/v) glycerol. The recombinant strain showed a notably high β‐xylosidase activity with a 13.8‐fold increment on day 6 of cultivation compared to Tam_WT as well as a markedly elevated endoxylanase activity, with a 22.7‐fold increase at day 4 of growth. In contrast, barely any of these activities were detected in Tam_WT (Figure 5A,B). Moreover, the measurements of extracellular protein concentration revealed that Tam_XlnR^V785F^ secretes more proteins than Tam_WT throughout the entire cultivation time (Figure S9C). Regarding the SDS‐PAGE and zymogram analyses (Figure 5C–E), similar results to those obtained with the glucose crudes were observed, although the differences in β‐xylosidase and endoxylanase abundance between Tam_XlnR^V785F^ and Tam_WT are not that pronounced in this case. As explained before, there are also variations in the band intensity pattern of Tam_XlnR^V785F^ depending on the substrate used, demonstrating that some level of regulation still remains. In the case of glycerol, the most intense bands in the endoxylanase zymogram (Figure 5D) are those around 50 and 20 kDa, while the 37 kDa one (the most abundant band in the xylan and glucose crudes) is barely detectable.

### 3.5 Enhanced Xylan Saccharification Using the Enzymatic Cocktails of Tam_XlnR^V785F^ Transformant

To assess the performance of the improved enzymatic cocktails produced by Tam_XlnR^V785F^ compared to those of Tam_WT, they were applied for the saccharification of beechwood xylan. Thus, Tam_WT and Tam_XlnR^V785F^ crudes were harvested from the cultures using different substrates when maximal hemicellulase activities were observed: after 4 days of growth in the case of xylan and glucose, and on day 6 of cultivation for glycerol. β‐xylosidase and endoxylanase activities, as well as extracellular protein concentrations of each crude used for the saccharification are displayed in Table 3. Since the improvement of hemicellulase activities is likely due to an increased concentration of the enzymes, the assays were conducted with equal volumes of the crudes.

**TABLE 3:** β‐xylosidase and endoxylanase activities, and extracellular protein concentrations of the enzymic cocktails from *T. amestolkiae'*s strains grown on Mandels medium supplemented with 1% (w/v) beechwood xylan and 1% (w/v) glucose for 4 days, and 1% (w/v) glycerol for 6 days.

Substrate	Strain	β‐xylosidase activity (U/L)	Endoxylanase activity (U/mL)	Extracellular protein concentration (mg/L)
Xylan *t* = 4 days	Tam_WT	998.42 ± 32.78	60.77 ± 6.84	198.97 ± 5.94
	Tam_XlnR^V785F^	3292.76 ± 26.38	236.41 ± 8.31	258.46 ± 1.94
Glucose *t* = 4 days	Tam_WT	44.67 ± 2.66	3.69 ± 0.67	112.46 ± 4.77
	Tam_XlnR^V785F^	876.84 ± 20.53	126.30 ± 4.8	156.68 ± 6.86
Glycerol *t* = 6 days	Tam_WT	68.70 ± 3.33	2.47 ± 0.18	136.74 ± 1.78
	Tam_XlnR^V785F^	945.25 ± 6.18	119.91 ± 4.99	158.62 ± 8.74

**FIGURE 6:**
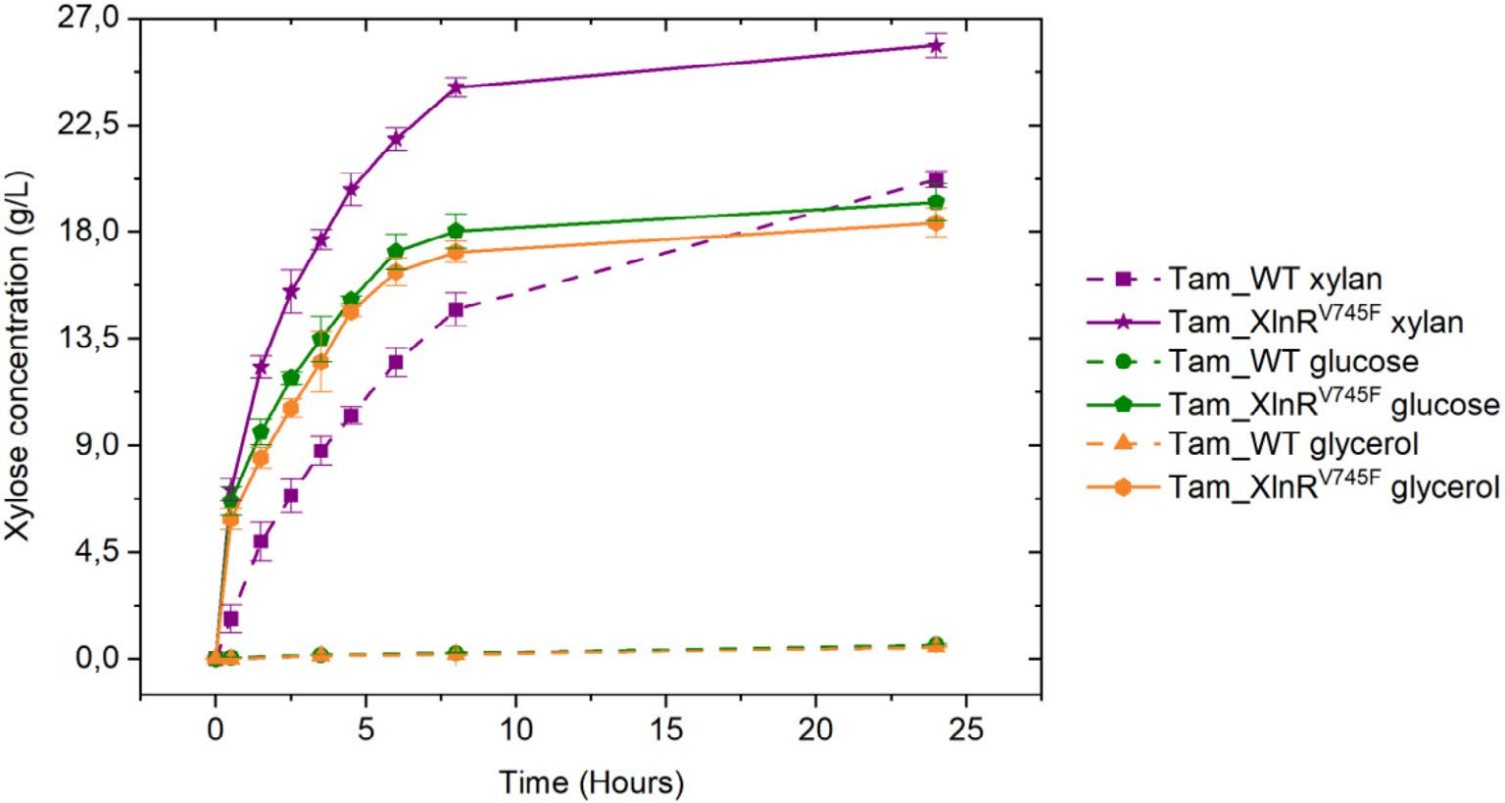
Saccharification of 50 g/L beechwood xylan with Tam_WT and Tam_XlnR^V785F^ enzymatic cocktails obtained after growing the fungi on Mandels medium supplemented with 1% (w/v) xylan, glucose and glycerol. Error bars represent standard deviations of three replicates.

Saccharifications were performed with 50 g/L xylan, applying a high agitation to ensure the homogeneity of the reactions, and they were monitored by measuring the release of xylose. The results of xylan saccharification with the different crudes of Tam_XlnR^V785F^ and Tam_WT can be found in Figure 6. For all the substrates used to obtain the crudes, Tam_XlnR^V785F^ reaches higher xylose concentrations than Tam_WT, demonstrating greater efficiency in producing hemicellulolytic enzymes, which leads to a more economically viable saccharification of plant biomass residues. This enhancement is even more pronounced for non‐inducing substrates, enabling the utilisation of low‐cost and renewable materials, such as glycerol, to produce these enzymatic cocktails, an outcome that would not be achievable with the wild‐type strain. Specifically, the saccharification yields after 24 h of Tam_XlnR^V785F^ and Tam_WT, respectively, were 51.7% and 40.4% for xylan crudes, 38.5% and 1.2% for glucose supernatants and 36.8% and 1.0% for glycerol cocktails.

## 4 Conclusions

A successful transformation procedure for the genetic manipulation of *T. amestolkiae* was developed, enabling the directed improvement of its biotechnological potential. Indeed, with this tool we demonstrated that forced expression of a Val785Phe mutant of the transcriptional activator XlnR is a promising strategy to increase hemicellulase production in this fungus, enhancing the efficiency of its hemicellulolytic cocktails for plant biomass valorisation. Furthermore, the Val785Phe substitution of XlnR in *T. amestolkiae* also leads to a partial deregulation of β‐xylosidase and endoxylanase expression under non‐inducing conditions. These results show the potential of genetic manipulation tools to design fungal strains that expand the biotechnological applications of *T. amestolkiae*.

## Author Contributions

**Ana Pozo‐Rodríguez:** conceptualization, methodology, writing – original draft, data curation. **Miguel Ángel Peñalva:** conceptualization, writing – review and editing. **Jorge Barriuso:** conceptualization, writing – review and editing. **Eduardo A. Espeso:** conceptualization, writing – review and editing, funding acquisition, supervision. **María Jesús Martínez:** conceptualization, funding acquisition, writing – review and editing, supervision.

## Conflicts of Interest

The authors declare no conflicts of interest.

## Supporting information


Data S1.

## Data Availability

Data are provided within the manuscript and S1 files.
