# Supplementary material for: Improvement of β‐Xylosidase and Endoxylanase Activities in Talaromyces amestolkiae by Genetic Manipulation of the Transcriptional Activator XlnR
Source: Microb Biotechnol. 2025 May 26;18(5):e70166. doi: 10.1111/1751-7915.70166 (PMC12105496; doi:10.1111/1751-7915.70166)

**Supporting information**

**Figure S1:** *De novo* pyrimidine biosynthetic pathway in fungi, which leads to the production of uridine 5′-monophosphate, the precursor of all pyrimidine nucleotides. The chart, on the right, includes enzymes and *A. nidulans* gene designations and, on the left, *T. amestolkiae* gene codes (genome available under the GenBank Accession Number ASM189636v1). Mutations incorporated by the acquisition of 5-FOA resistance will be found in the last two steps, prior to UMP biosynthesis (*pyrF* and *pyrG* genes).


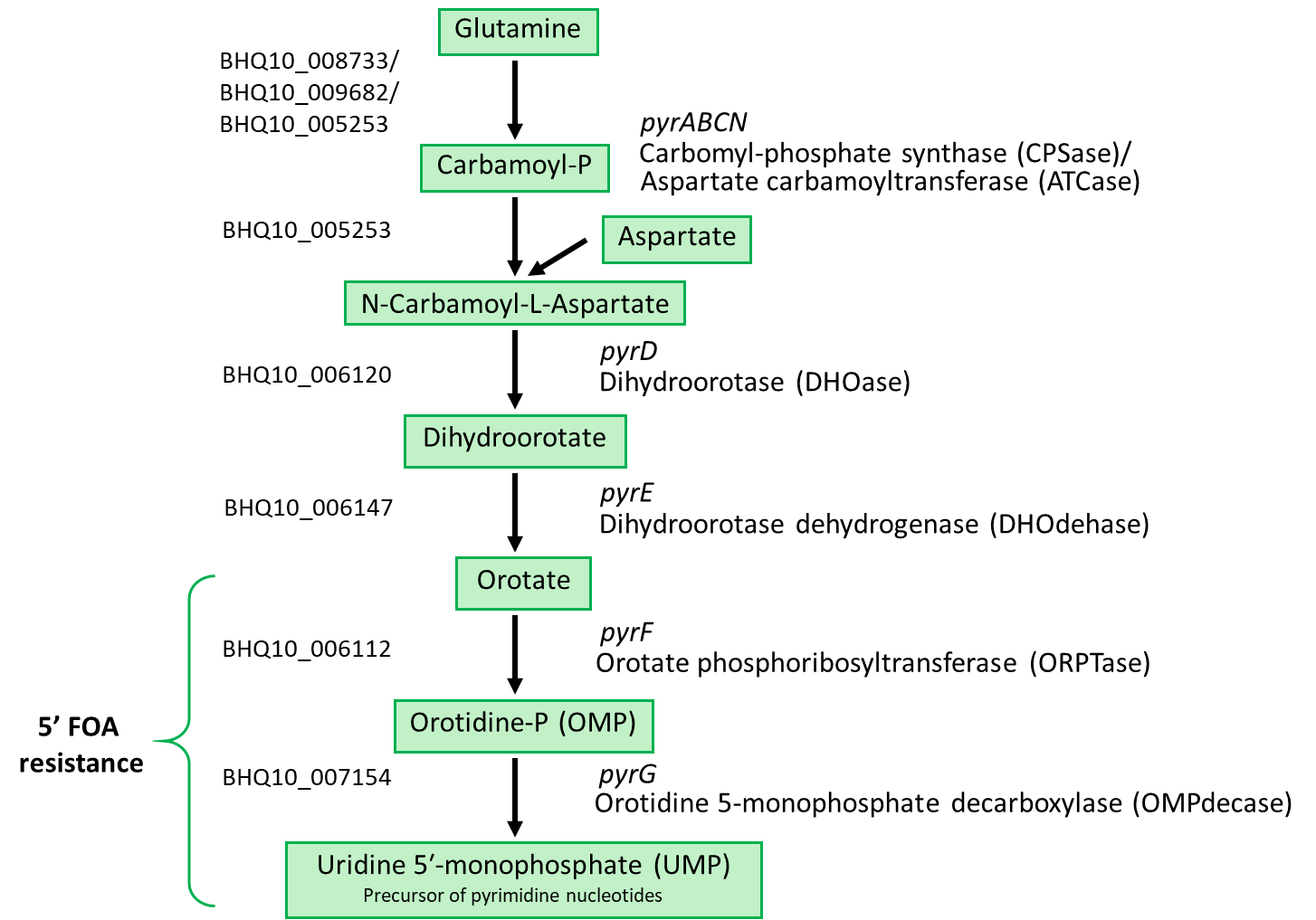


**Table S1:** Primers used in this study.

| **Primers** | **Sequence** | **Use** |
| --- | --- | --- |
| Seq_pyrG_fw | ttccttccaactcaatcaag | Sequencing of *T. amestolkiae pyrG* |
| Seq_pyrG_rv | tagggcttggtgtcccctca |  |
| Seq_pyrF_fw | atttattccgcagtattccg | Sequencing of *T. amestolkiae pyrF* |
| Seq_pyrF_rv | cgtgactatcgcatctcagc |  |
| p1439_fw | ccagattggcacgcaatttttac | Genotyping PCR of Tam_p1439 transformants |
| p1439_rv | gagaggttcacacccgtcgtg |  |
| Seq_p1393-PgpdA-XlnR_1 | ggatgtgctgcaaggcgatt | Sequencing of PgpdA_XlnR_TtrpC insert and its mutated derivatives Cloning in p1393 and genotyping PCR of Tam_XlnR, Tam_XlnR^A788V^ and Tam_XlnR^V785F^ transformants |
| PCR/Seq_p1393-PgpdA-XlnR_2 | ctttgctacatccatactcc |  |
| Seq_p1393-PgpdA-XlnR_3 | agcgactgcggaatgatcg |  |
| Seq_p1393-PgpdA-XlnR_4 | gtgctagagaccatgctgca |  |
| Seq_p1393-PgpdA-XlnR_5 | tgccgtatgcgacgaccatt |  |
| PCR/Seq_p1393-PgpdA-XlnR_6 | gctgacatcgacaccaac |  |
| Seq_p1393-PgpdA-XlnR_7 | cgatccacttaacgttactg |  |
| PCR_p1393_fw | cgtaaggagaaaataccg | Genotyping PCR of Tam_p1393 transformants |
| PCR_p1393_rv | tcactggccgtcgttttac |  |
| PCRmut_XlnR_A788V_fw | gccacgccgtcaaggccgtcgaagcagcgtccgat | Mutagenic PCR to obtain XlnR^A788V^ |
| PCRmut_XlnR _A788V_rv | atcggacgctgcttcgacggccttgacggcgtggc |  |
| PCRmut_XlnR _V785F_fw | cagctatgggccacgccttcaaggccgccgaagc | Mutagenic PCR to obtain XlnR^V785F^ |
| PCRmut_XlnR _V785F_rv | gcttcggcggccttgaaggcgtggcccatagctg |  |
| SeqMut_XlnR | gcacgttttacatattctgc | Sequencing XlnR^A788V^ and XlnR^V785F^ mutations |

**Figure S2:** T. amestolkiae XlnR (GeneBank Accesion Number BHQ10_006915). A) Nucleotide sequence of T. amestolkiae xlnR (1-2858 nt). Introns are displayed in blue. Silent mutations were introduced for cloning reasons and are highlighted in red. Codons of A788 and V785 residues are indicated in yellow and green, respectively, and the nucleotide substituted for A788V and V785F mutations are in bold. B) Amino acid sequence of T. amestolkiae XlnR (1-904 aa). A788 and V785 residues are depicted in yellow and green, respectively. C) Structural model predicted by Alphafold. The Zn_2_Cys_6_ DNA-binding domain (85-128 aa, orange) and the middle homology region (Fungal_TF_MHR, 347-807 aa, blue) were predicted by InterProScan. A788 (yellow) and V785 (green) residues form part of the same α-helix (purple) and are located in the middle homology region near the C-terminal end of the protein.


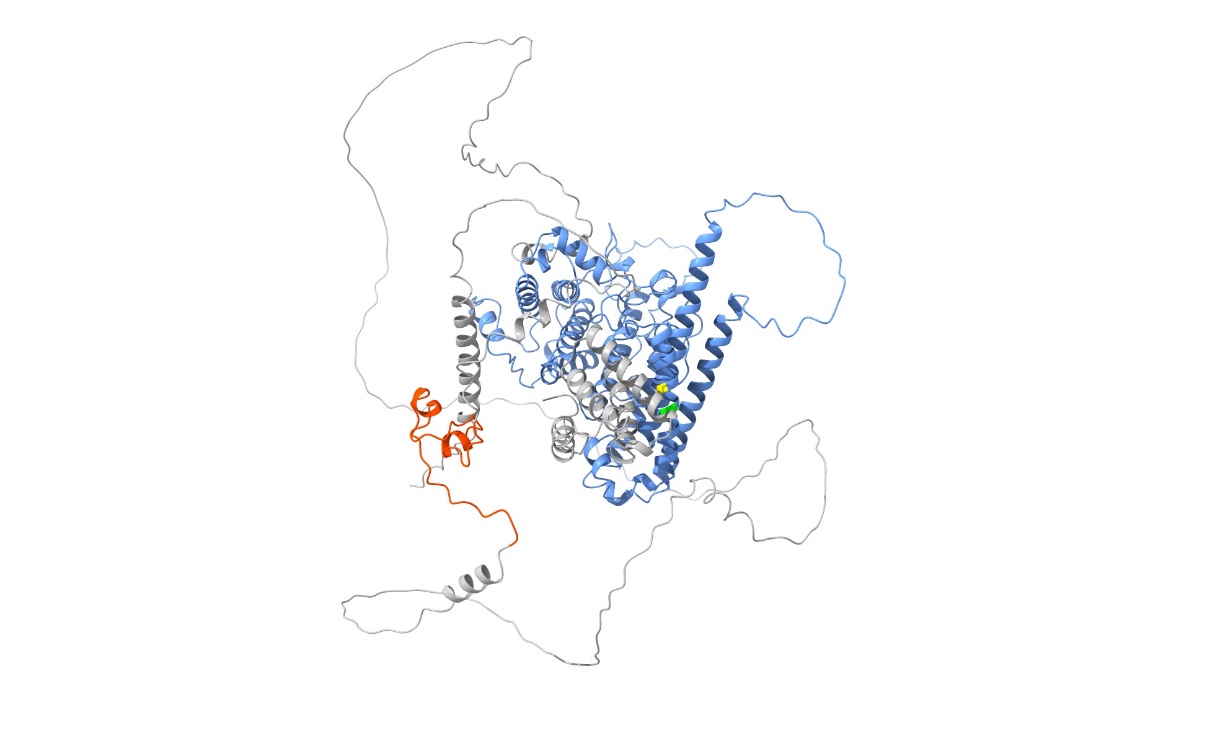


**C-term**

**N-term**


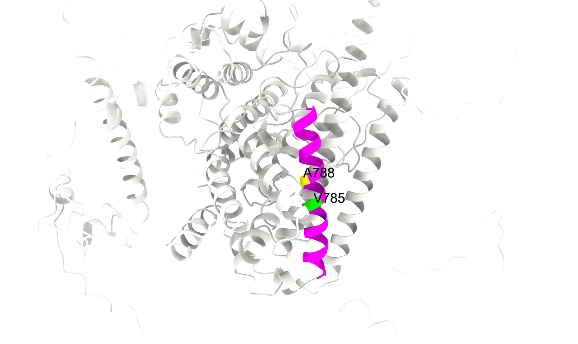


**A)**

MAQPSQTPGLDTLAESSHYALEQMRLAREVDMNNSSSSNGNNDFVKKDNKAIADSGSRMQIMRSPLSDTRAGIRKHSADTAAVRRRISRACDQCNQLRTKCDGQSPCAHCVDSGLSCEYARERKKRGKASKKDIAEAAAKAGGGAREPGTPGYETVPDQSSQLSAVMASESDARLSQSRRSFSASQVADQQPGLASLRELAQQQQQQQQPSQTRSRQFFPSNMNSMTMNGYGQVQNVDRSFIQMPDLRDLQPRSPSAIVPVGLNGFHDAYMVDHAPTNMNQYQYPQSGEENTANHFTGLTPPVQSPGWLPLPAPSVGFPSLNMSNGFTSTLKYPVLEPLLPHIVSIIPQSLACDLLDLYFASTSASHIFPQSPYVVGHVFRKKSILHQTQPRTCSPALLASMLWVAAQTSDAPFLTSPPSARGRVCQKLLELTVGLLRPLIHGPTPGETSPNYAANAVINGVALGGFGVSMDQLGAQSSATGAVDDVATYIHLATVVSASEYKAASIRWWAAAWSLARELKLGRELPPTPSQPQNHDRDGTIEIEPKPTRDTSHVTEEEREERRRIWWLLYVMDRHLALCYNRPLTLLDKECEGLLQPMNDDIWHRGELVNAGYRRAGPSFECTGHGMFGYFLPLMTILGEIVDLNHARNHPRFGIHFRTSGEWDSHTVEITRQLDVYEQSLREFETRHTASLGIGNEGAADAGFNATAPTGIDHVSPSARSSSTVGSRVNESLMQTKMVVAYGTYLMHVLHILLVGKWDPISLLDDNDLWISSEAFITAMGHAVKAAEAASDILEYDPDLSFMPFFFGIYLLQGSFLLLLTADKLQGDADPSVVRACETIVRAHEACVVTLNTEYQRNFRKVMRSALAQVRGRVPDDFGEQQQRRREVLALYRWTGDGSGLAL*

**B)**

atggcacaaccgtcgcagacacccggcttggacactctcgccgagagttcgcattacgctctggagcaaatgcgtcttgcgcgcgaagtcgatatgaacaacagcagtagcagcaacggcaacaatgatttcgtcaaaaaggataataaggccattgcggattctggctcaagaatgcagattatgcgcagtccgctttcagatacgagggctggtattcgcaagcattctgctgatacggcggcggtgcggcgccggattagtcgagcttgcgatcagtgtaatcagctacgaacgaagtgtgatgggcagagtccttgtgcacattgcaccggtgagatagaatgtcacatacaaatttaataaattgctgaggctaattcaacatcttttcgggtcggtagattccggtctgagttgcgaatatgctagagaaagaaaaaagcgcggaaaagcatcaaaaaaagatatcgcagaagccgctgcgaaagctggtggtggtgctcgcgaacctggtactcccggctacgagacggttccagatcagtcatctcaattgtcggcggtcatggcctcagagtctgatgcgcggctgagtcagtcgagacgctcgttttcagcgtcgcaggtcgcagaccaacaaccgggtcttgctagtctgcgcgaacttgcgcaacagcagcaacagcagcagcagccatcacaaacgagatcaagacagttcttcccatcgaacatgaattcaatgacgatgaatggatatggacaggtgcagaatgtagatcgatcgttcattcagatgcctgatctgcgagacttgcagccgaggtcgccatctgcgatagttccggttgggttaaatgggtttcacgatgcgtacatggttgatcacgcgccgaccaatatgaatcaatatcaatacccacagtctggagaagaaaatacagcaaaccactttactggcctcactcctcctgtccagtcgcccggatggttacccttgcctgcgccatccgtggggtttccatcgctgaatatgtcgaatggtttcactagcacattgaaatatcccgtgctagagccattgttacctcatattgtttcgatcattccgcagtcgctggcgtgtgatttgctcgacctctactttgccagcacgtctgcctcacatatctttccccagtcaccgtacgtggtaggacacgtattccgaaagaagtcgatcctacatcaaactcaaccacgcacatgcagtcccgctttattggcgagcatgctctgggtagcagctcagactagcgatgcgccgtttttgacatcacctccgtcagcgcgaggaagggtctgtcagaagttactggagcttacagttggcttgctacgacccttaatacatggtcctacgcccggagaaacgtcgcctaattacgctgccaatgcggtcatcaacggagttgcgctaggcggattcggtgtatctatggatcagctcggtgcacaaagcagcgctaccggagcagtcgacgacgttgccacatacattcacctagcgactgtggtatcagccagcgagtacaaggctgcaagcatccgctggtgggctgcagcatggtctctagcacgcgaactaaaactcggacgtgaacttccgccaacgccctcgcagcctcagaatcatgaccgagatggcactattgagatagagcccaaaccgacgcgagatacgagccatgtcaccgaagaagaacgagaagaacgtaggcgaatatggtggcttctctacgtgatggatcgccatttggcgttatgctacaaccggccgttgacattactggataaagagtgtgaagggttgctacagccgatgaacgacgatatttggcacagaggcgagcttgtgaatgccggttaccgacgagcaggtcccagctttgaatgtacaggccacggcatgtttgggtacttcctaccgttgatgactattcttggtgaaatcgtcgatttgaatcatgcgcggaaccatcctcggtttggtattcattttcgaacgagcggcgagtgggatagtcatactgtggagattactcgacagctcgatgtctacgaacagagtctacgcgagtttgagactcgacatacggcgagtttgggcattggaaatgagggcgctgctgatgctggattcaatgctacagcacccacagggattgatcatgtcagtccatcagcgagatcgtcgagcacagtgggctcccgagtcaatgaatcgcttatgcagaccaaaatggtcgtcgcatacggcacctacctcatgcacgttttacatattctgctcgtcggaaagtgggacccgatctctttactagacgataacgatctctggatttcatcagaagccttcatcacagctatgggccacgcc**g**tcaaggccg**c**cgaagcagcgtccgatatattagaatacgacccagatttgagttttatgccattcttcttcggcatttatctcctacaaggcagtttcctgctactgcttacggccgataaattacaaggcgacgctgaccctagcgttgtcagggcatgcgagacgatagtacgggcgcacgaagcgtgcgttgtaacactgaatacagagtatcaggttcgtcattgtgtccctttagcctattctcccccaatttttttttttagttatattgctgacgacgttgcagcgcaatttccgaaaagtcatgcgttccgcactggcacaagtccgcggacgcgtcccagacgattttggcgaacagcaacaacgacggcgcgaggttctcgcactttatcgctggaccggtgacggcagcggattggctttatga

**C)**


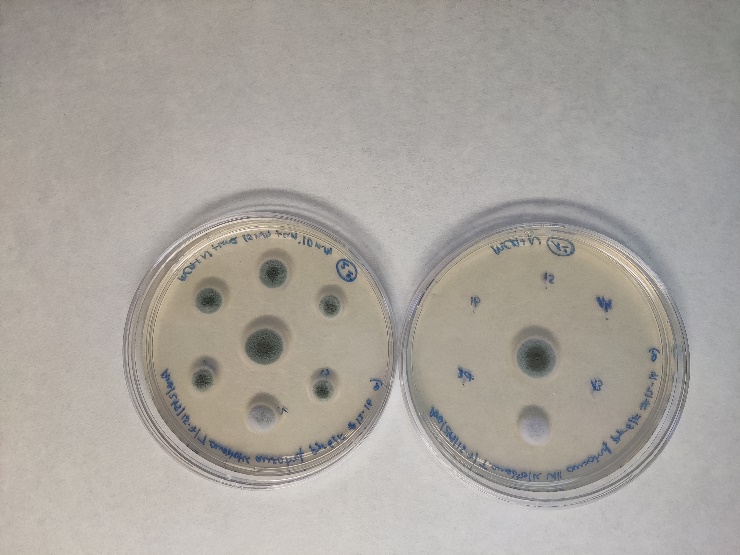


WT

#12

#3

#16

#15

#14

#13

#14

#13

#3

#16


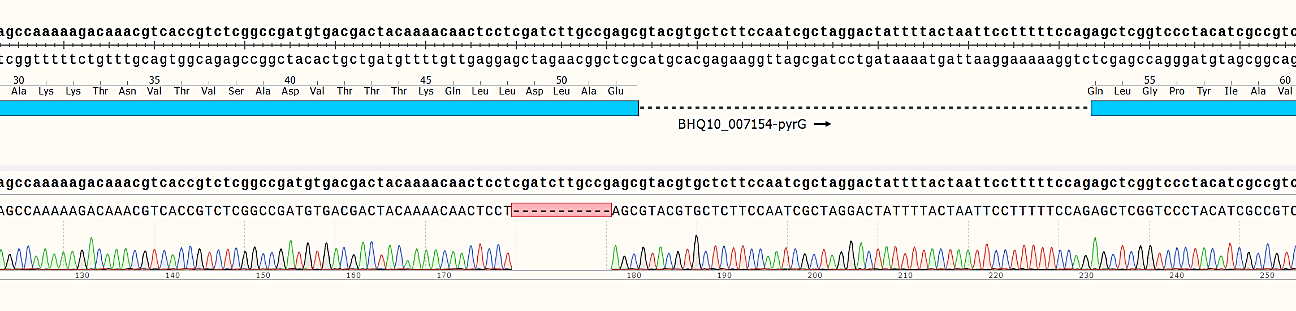


WT

#12

#13

#12

#13

#14

#14

#15

#15x

#16

#3

#3

**A)**

**B)**

**MCA**

**MCA + uri + ura 10 mM**

**Figure S3:** *T. amestolkiae pyr*- auxotrophs. A) Growth of the wildtype strain and potential *pyr*- mutants (#3,#12-#16), obtained through the acquisition of 5’fluoorotic acid (5’FOA) resistance, in MCA plates with and without uridine and uracil. Most of the mutants showed the expected auxotrophic phenotype, requiring pyrimidine supplementation for their growth, except #12 that was able to grow in the absence of uridine and uracil, indicating that it is not a *pyr^-^* auxotroph. B) Genotype of Tam_*pyrG*14 auxotroph harbouring the c.Δ(C144→G154) mutation.

#12

#15


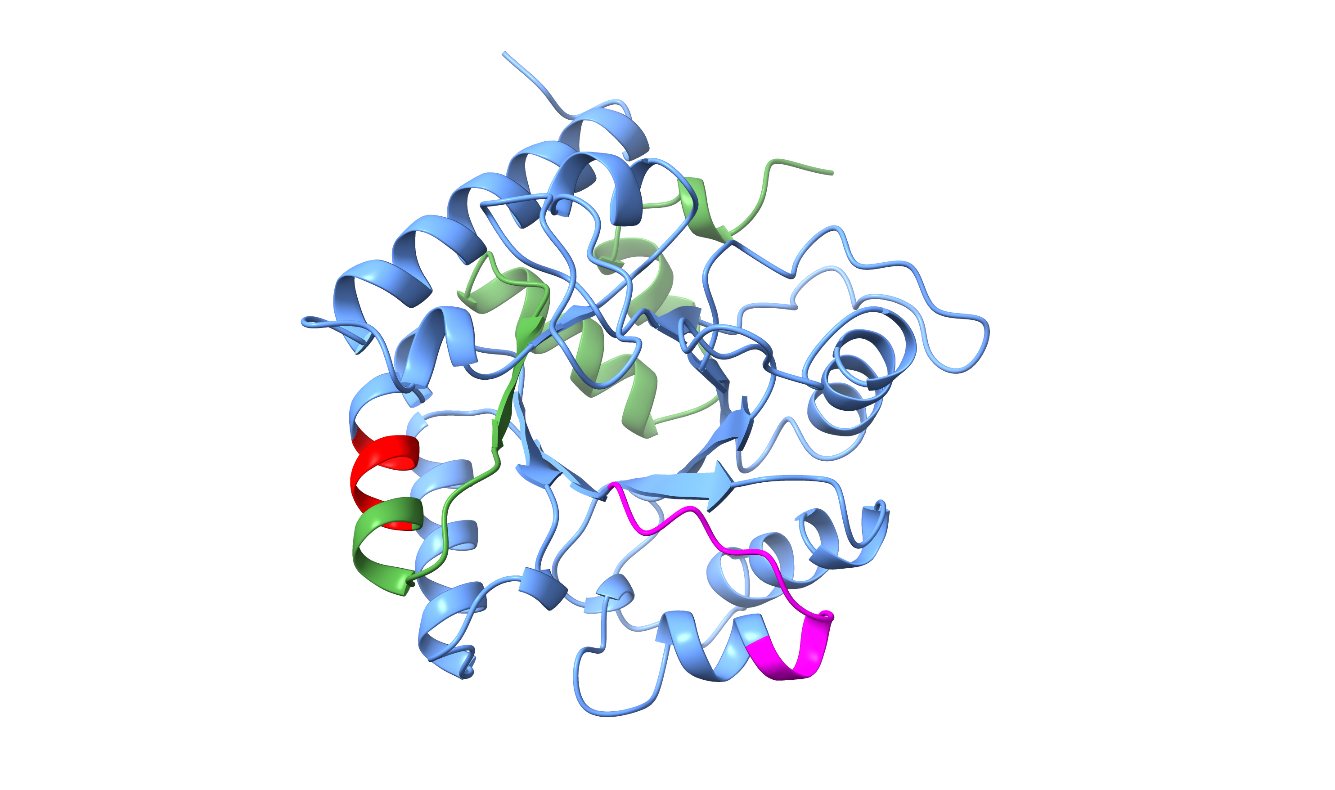


**Figure S4:** T. amestolkiae orotidine 5-monophosphate decarboxylase (OMPdecase, 1-278 aa) encoded by pyrG (GeneBank Accesion Number BHQ10_007154). OMPdecases are normally dimeric. The structural model was predicted by Alphafold and depicts only one of the monomers. The protein change derived from the c.Δ(C144→G154) mutation of Tam_pyrG14 is shown in different colours: in green the unaltered amino acids of the N-terminal end, in red the deleted residues D49-E52 and in blue the rest of the truncated sequence. The truncation begins after L48, which is located upstream of the main catalytic residues (DXKXDIXXT) highlighted in purple, strongly indicating a complete loss-of-function of OMPdecase.

**N-term**

**C-term**


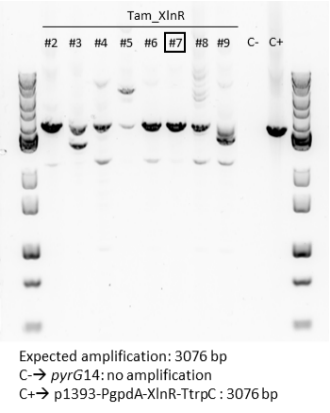

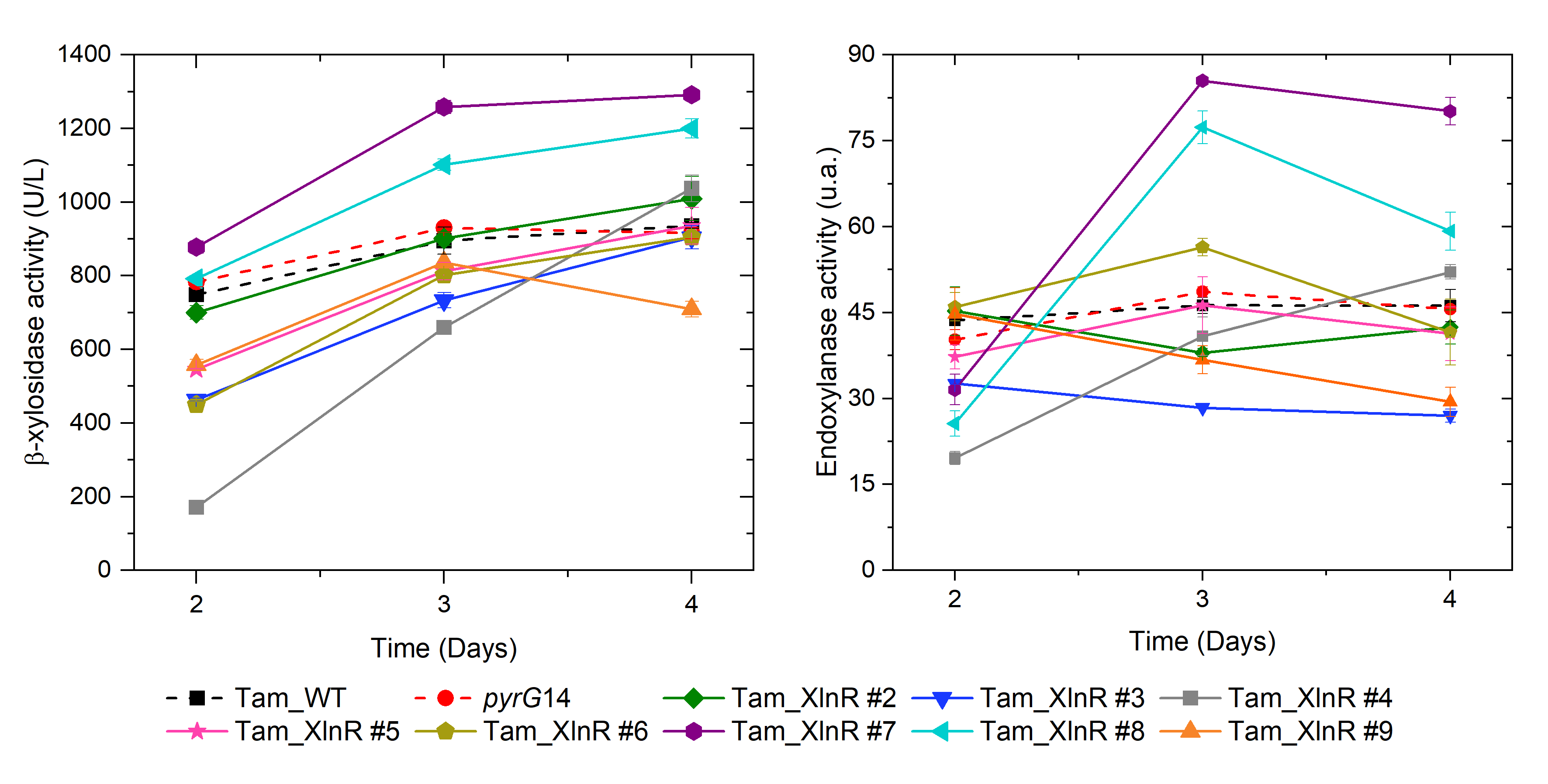


**Figure S5:** Screening of the best performing mutant (highlighted with a black square) of Tam_*pyrG*14 transformation with p1393-PgpdA-XlnR-TtrpC plasmid (Tam_XlnR). The screening was conducted with 8 transformants randomly selected. A) β-xylosidase and B) endoxylanase activities of the supernatants from Tam_XlnR transformants grown in Mandels medium supplemented with 1% (w/v) beechwood xylan. Tam_WT and Tam_*pyrG*14 were included as controls. C) Genotyping PCR of Tam_XlnR transformants. Tam_*pyrG*14 was used as negative control and the plasmid as positive control. 1 kb DNA Ladder Plus NIPPON Genetics was employed.

**B)**

**A)**

**C)**

3 kb


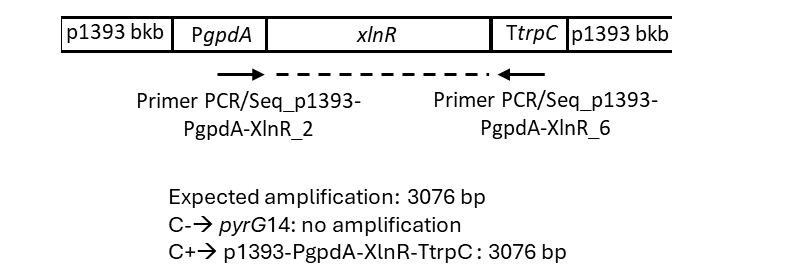

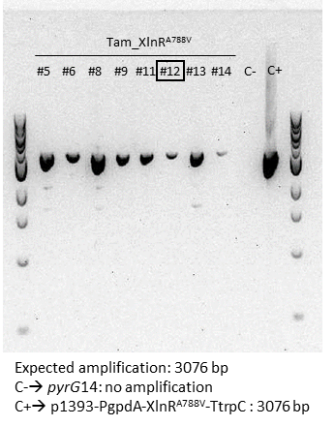

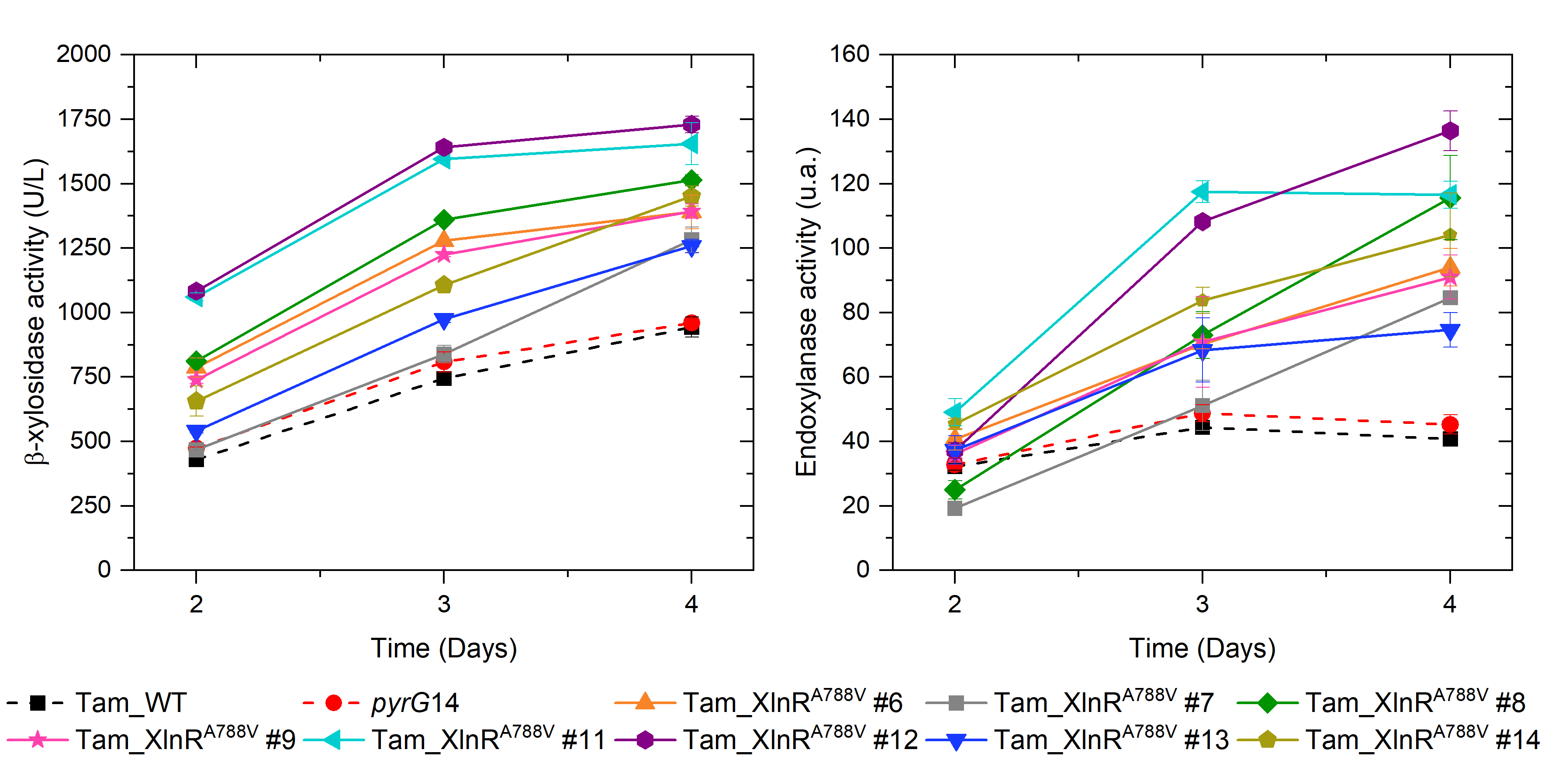


**Figure S6:** Screening of the best performing mutant (highlighted with a black square) of Tam_*pyrG*14 transformation with p1393-PgpdA-XlnRA788V-TtrpC plasmid (Tam_XlnRA788V). The screening was conducted with 8 transformants randomly selected. A) β-xylosidase and B) endoxylanase activities of the supernatants from Tam_XlnRA788V transformants grown in Mandels medium supplemented with 1% (w/v) beechwood xylan. Tam_WT and Tam_*pyrG*14 were included as controls. C) Genotyping PCR of Tam_XlnRA788V transformants. Tam_*pyrG*14 was used as negative control and the plasmid as positive control. 1 kb DNA Ladder NEB was employed.

**C)**

**A)**

**B)**

3 kb


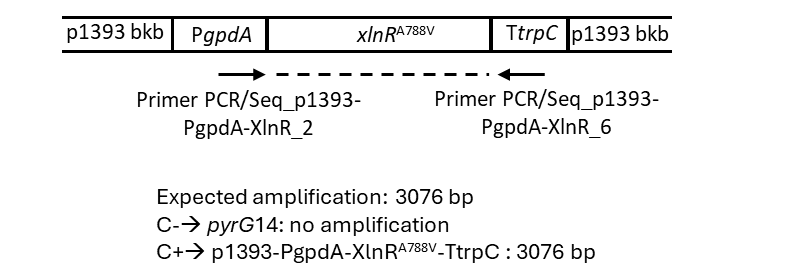

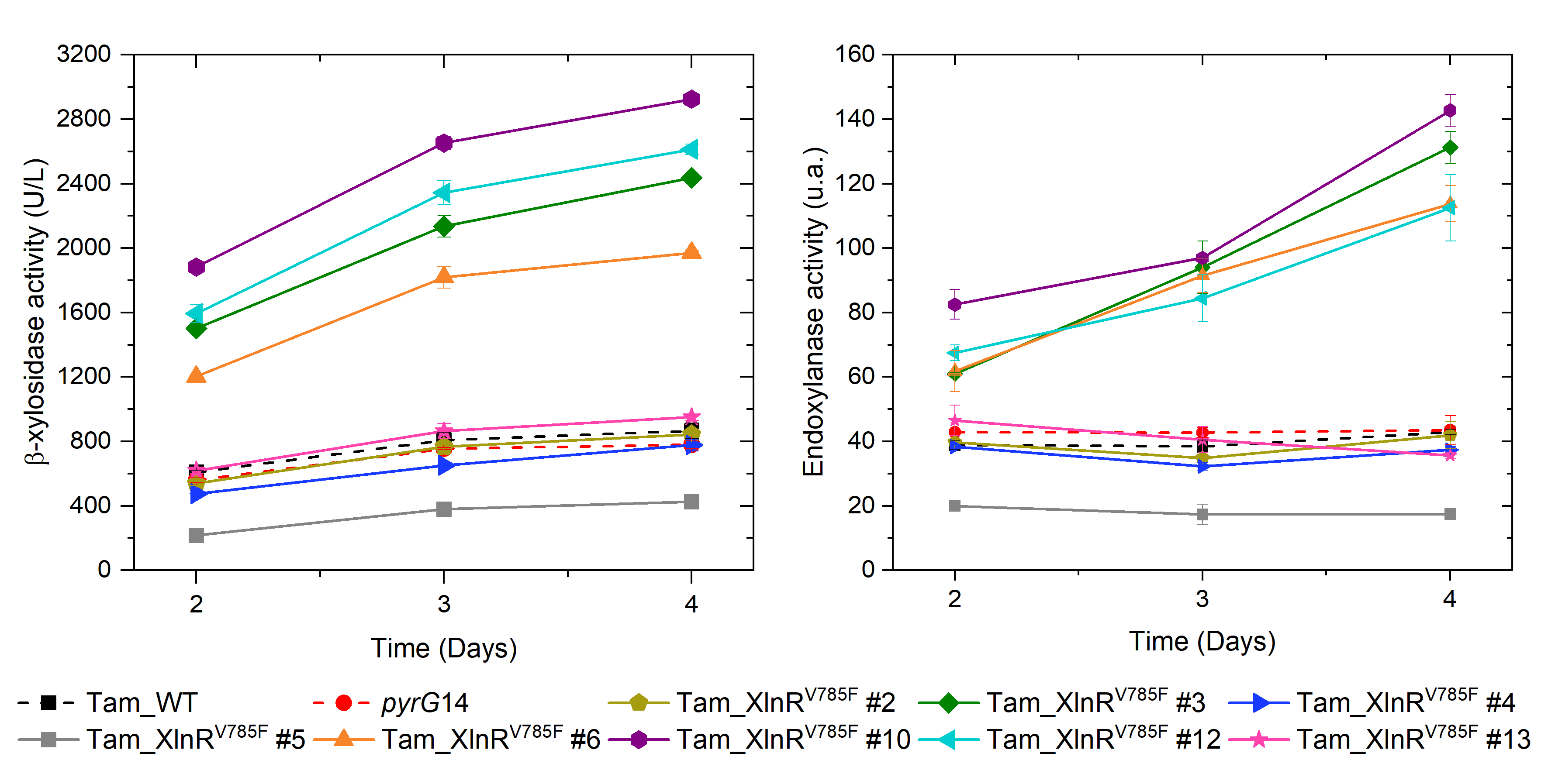

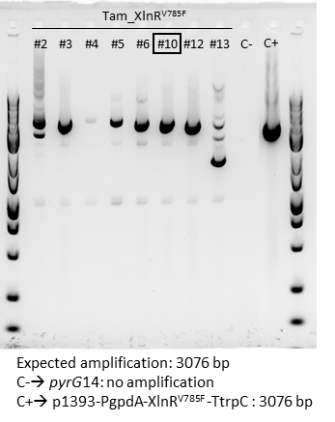


**Figure S7:** Screening of the best performing mutant (highlighted with a black square) of Tam_*pyrG*14 transformation with p1393-PgpdA-XlnRV785F-TtrpC plasmid (Tam_XlnRV785F). The screening was conducted with 8 transformants randomly selected. A) β-xylosidase and B) endoxylanase activities of the supernatants from Tam_XlnRV785F transformants grown in Mandels medium supplemented with 1% (w/v) beechwood xylan. Tam_WT and Tam_*pyrG*14 were included as controls. C) Genotyping PCR of Tam_XlnRV785F transformants. Tam_*pyrG*14 was used as negative control and the plasmid as positive control. 1 kb DNA Ladder Plus NIPPON Genetics was employed.

**C)**

**A)**

**B)**

3 kb


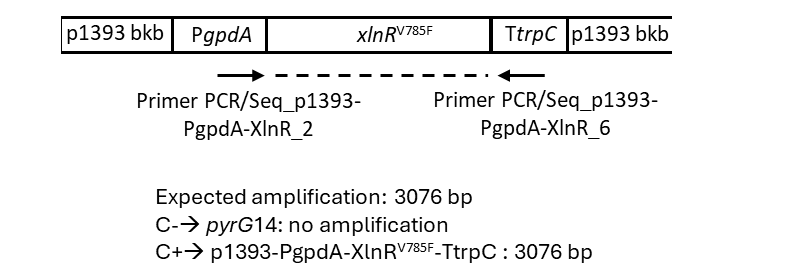

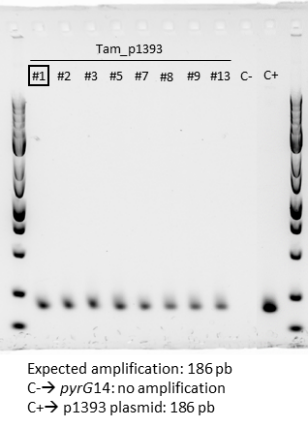

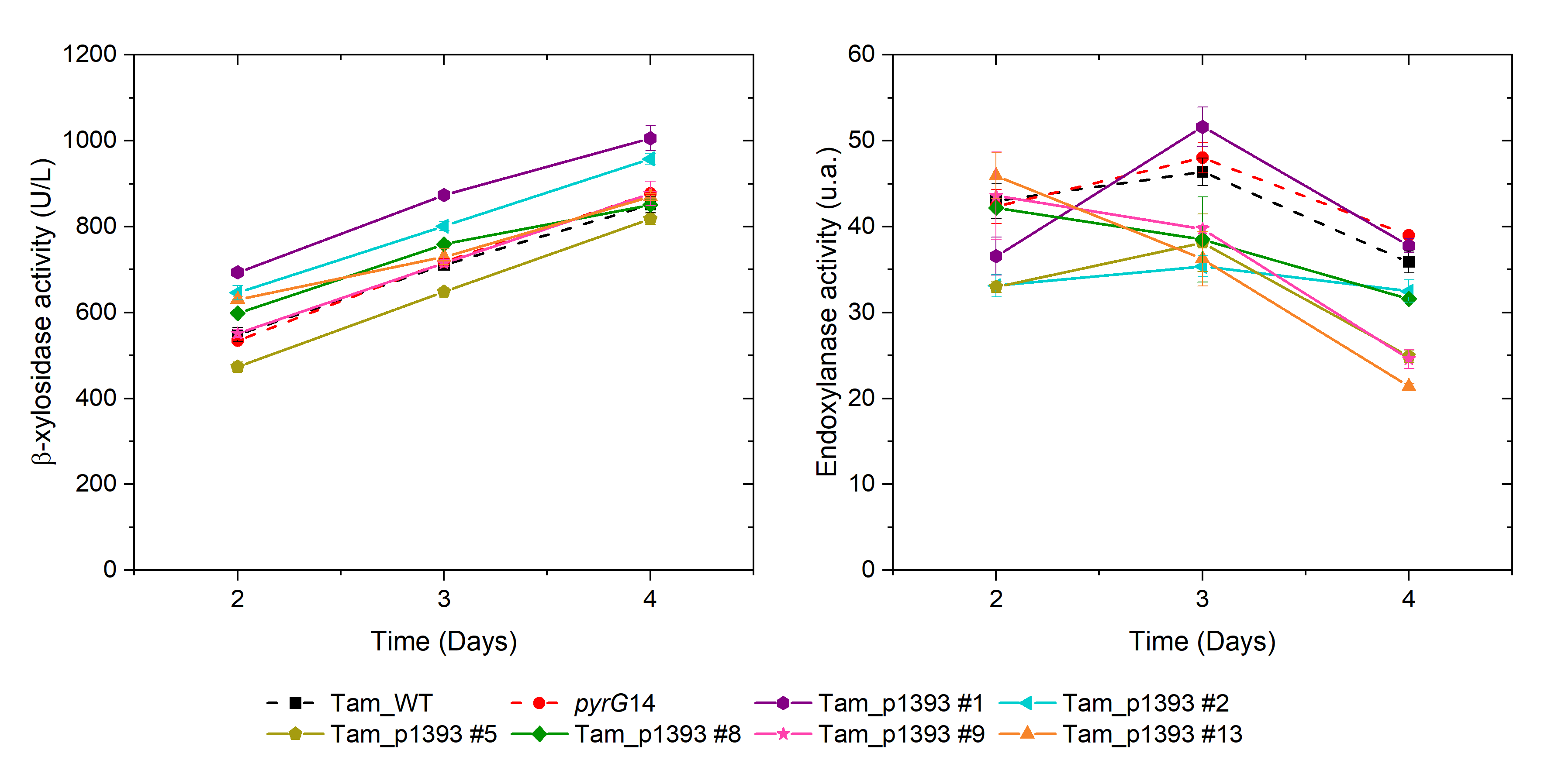


**Figure S8:** Screening of the best performing mutant (highlighted with a black square) of Tam_*pyrG*14 transformation with p1393 autonomously replicating plasmid (Tam_p1393). The screening was conducted with 6 transformants randomly selected. A) β-xylosidase and B) endoxylanase activities of the supernatants from Tam_p1393 transformants grown in Mandels medium supplemented with 1% (w/v) beechwood xylan. Tam_WT and Tam_*pyrG*14 were included as controls. C) Genotyping PCR of Tam_p1393 transformants. Tam_*pyrG*14 was used as negative control and the plasmid as positive control. 1 kb DNA Ladder Plus NIPPON Genetics was employed.

**A)**

**B)**

**C)**

250 bp


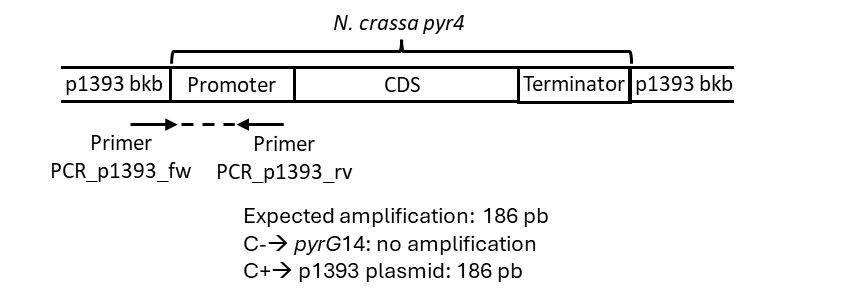

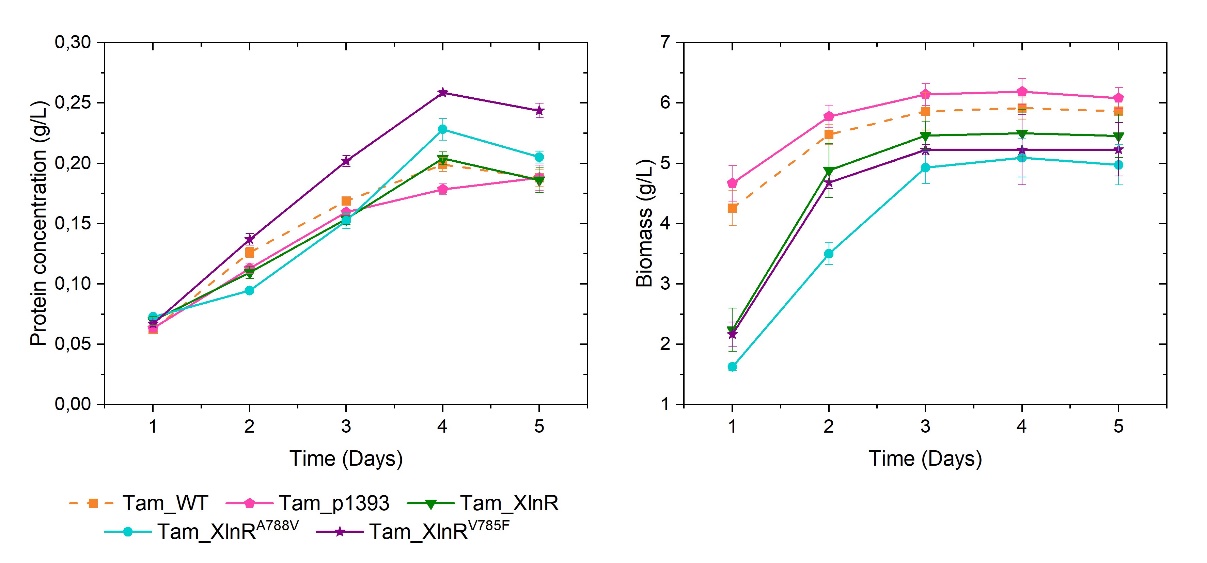


**Figure S9:** Extracellular protein concentration of *T. amestokiae*‘s strains when cultivated in Mandels medium supplemented with different carbon sources. A) Tam_XlnR, Tam_XlnRA788V, Tam_XlnRV785F, Tam_p1393 and Tam_WT with 1% (w/v) beechwood xylan. B) Tam_XlnR, Tam_XlnRV785F and Tam_WT with 1% (w/v) glucose. C) Tam_XlnRV785F and Tam_WT with 1% (w/v) glycerol. Error bars represent standard deviations of 3 biological replicates.


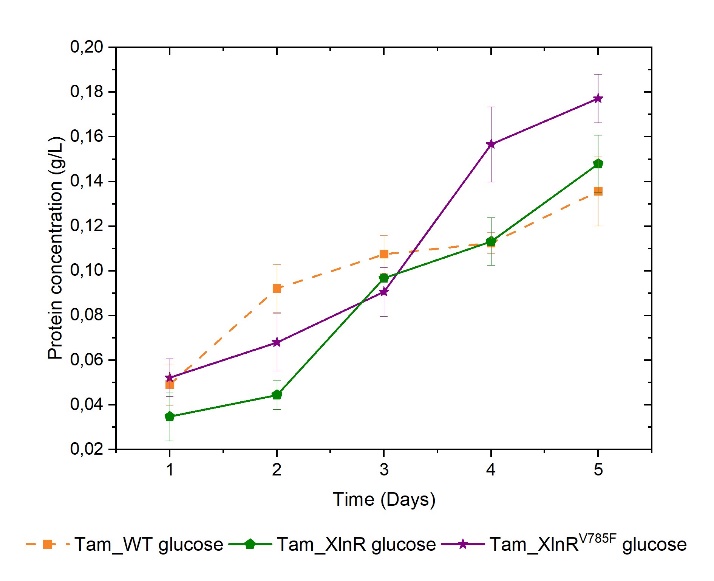

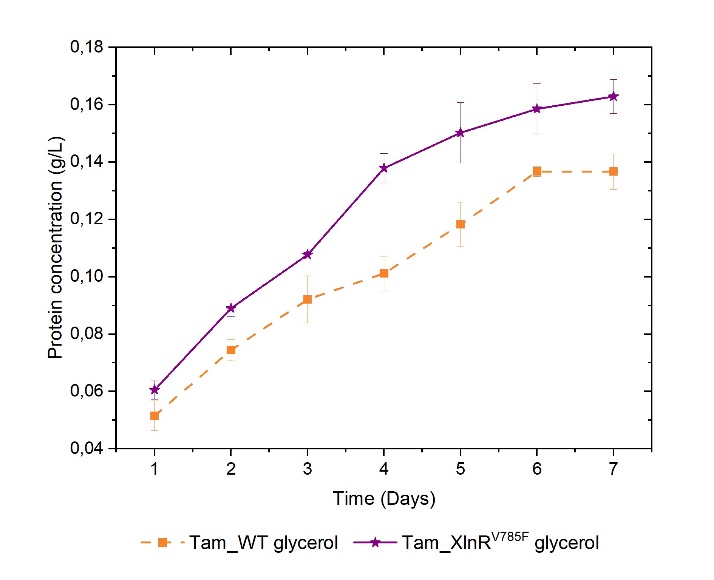


**A)**

**B)**

**C)**


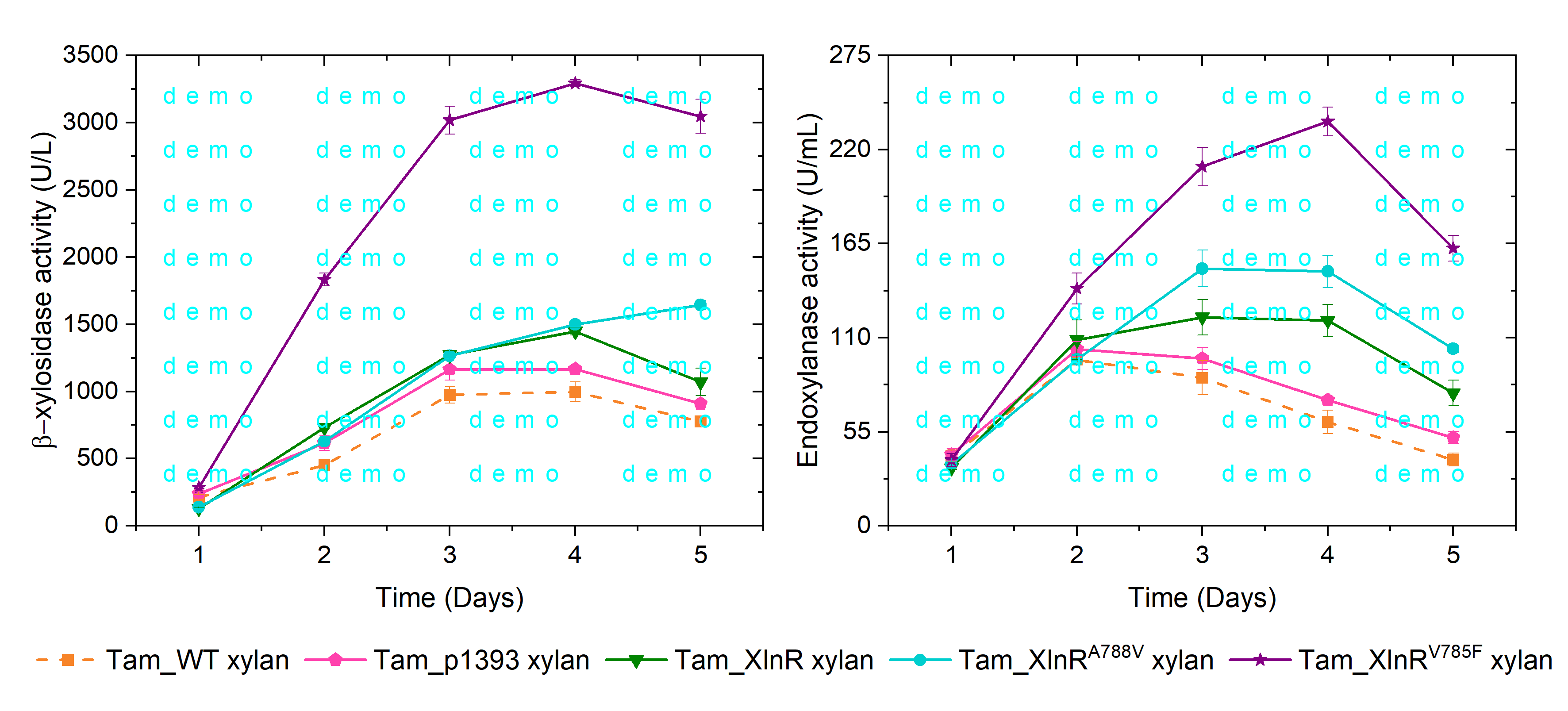

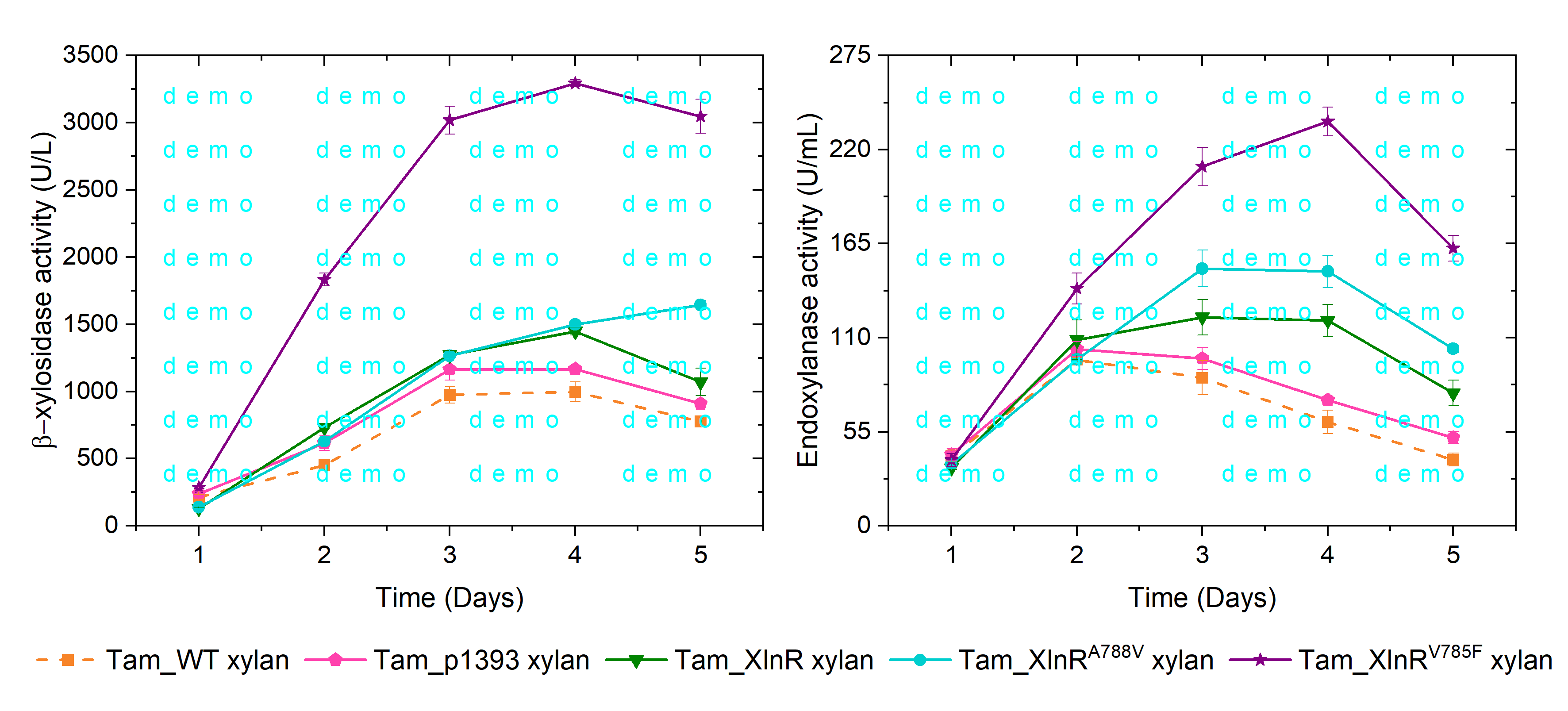

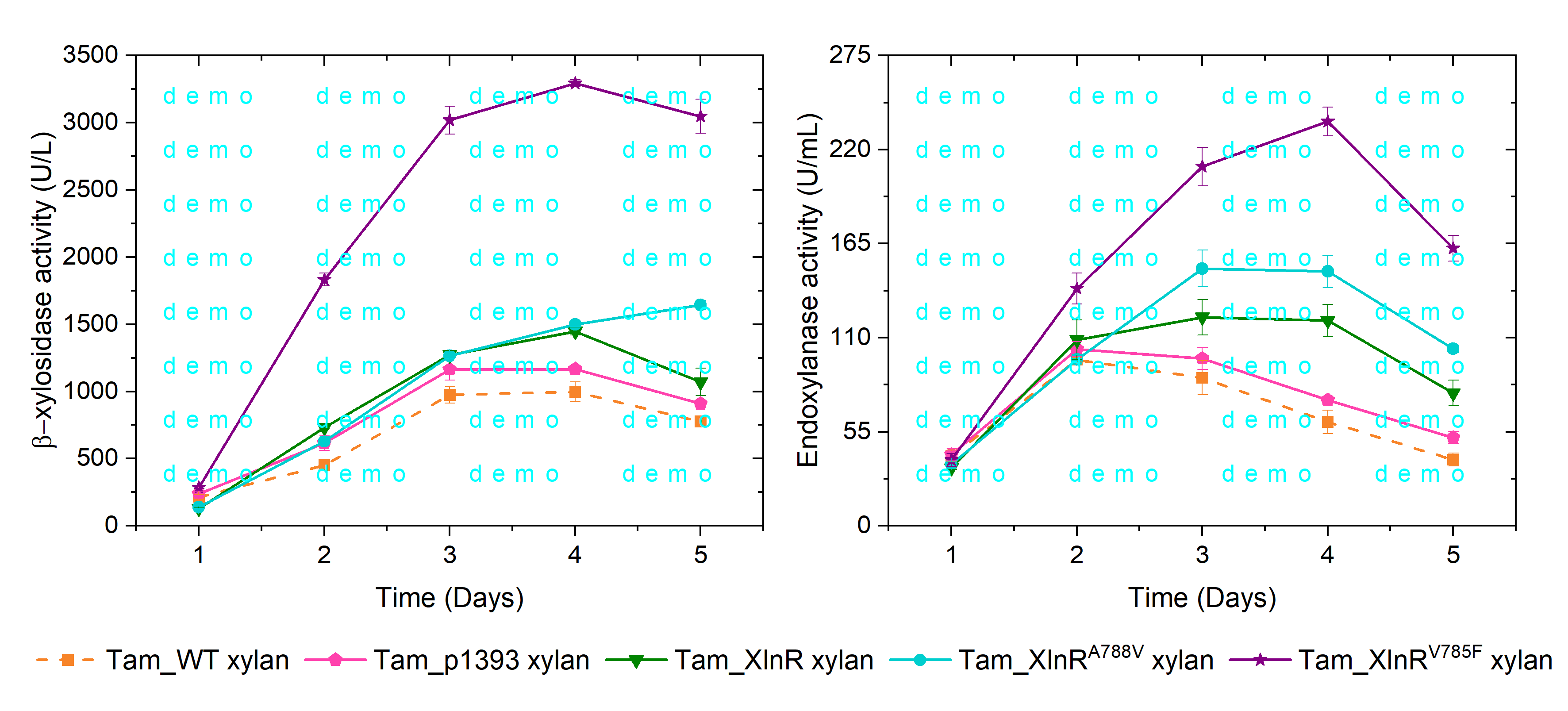

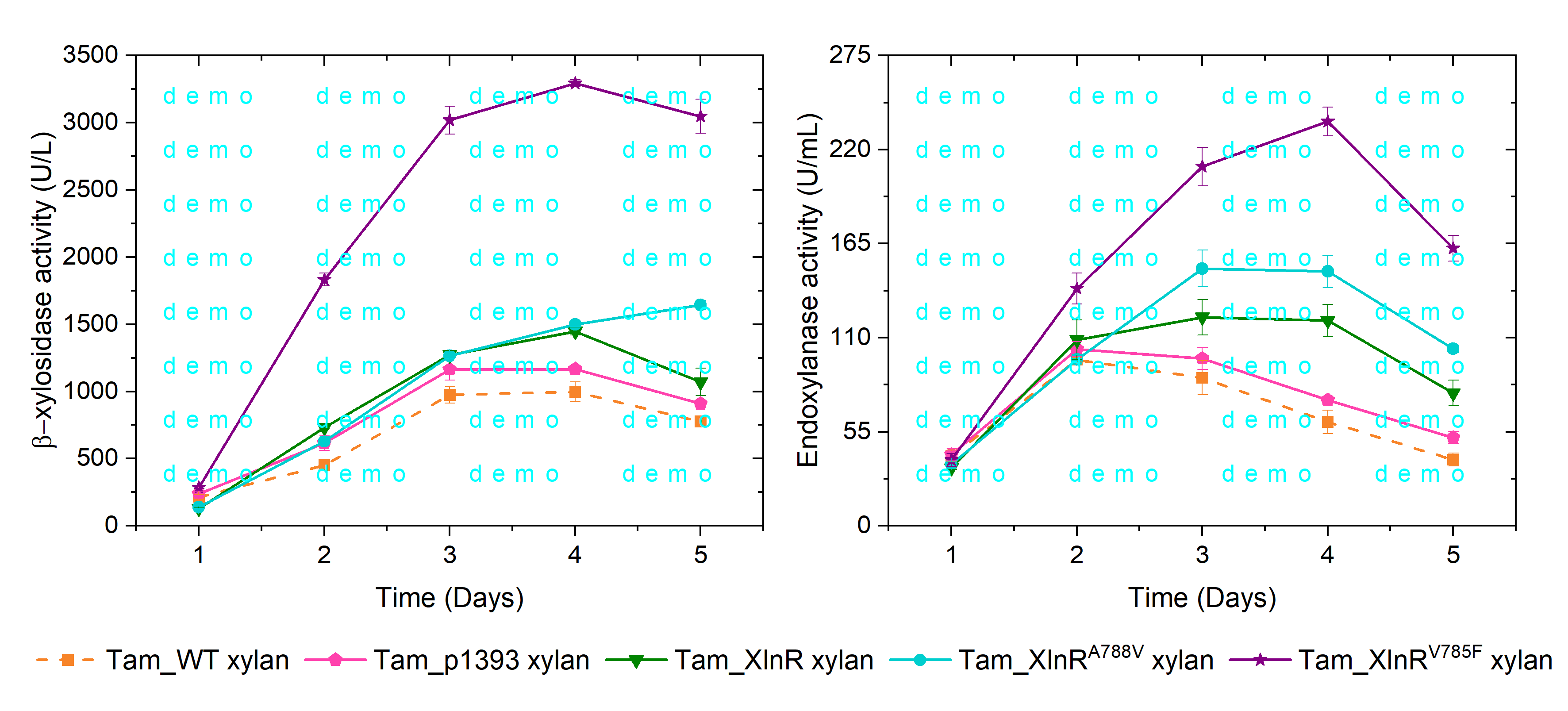

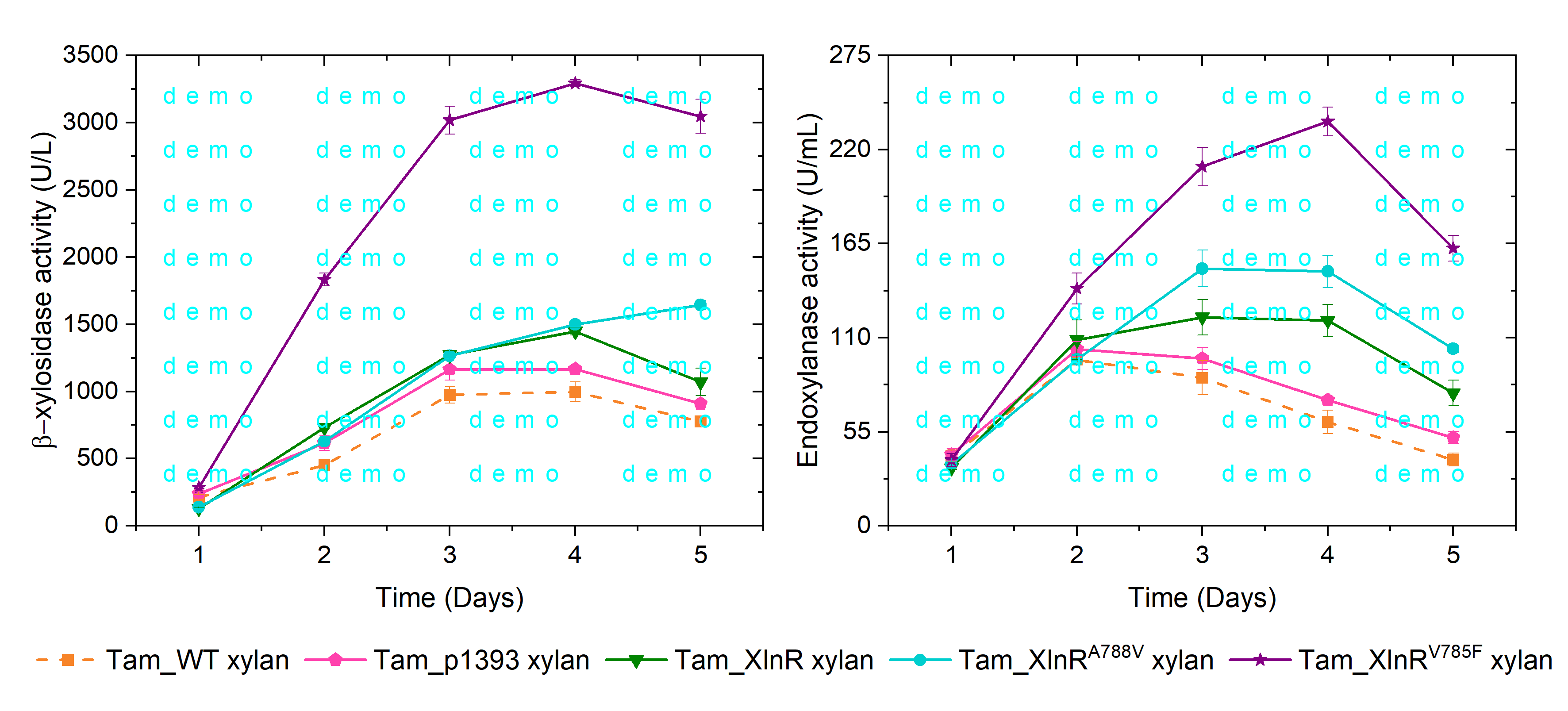

Supplement: Supplementary file 1 — Data S1. [file MBT2-18-e70166-s001.docx]
